# Discovery of triterpenoids as potent dual inhibitors of pancreatic lipase and human carboxylesterase 1

**DOI:** 10.1080/14756366.2022.2029855

**Published:** 2022-01-31

**Authors:** Jing Zhang, Qiu-Sha Pan, Xing-Kai Qian, Xiang-Lu Zhou, Ya-Jie Wang, Rong-Jing He, Le-Tian Wang, Yan-Ran Li, Hong Huo, Cheng-Gong Sun, Lei Sun, Li-Wei Zou, Ling Yang

**Affiliations:** a Institute of Interdisciplinary Integrative Medicine Research, Shanghai University of Traditional Chinese Medicine, Shanghai, China; b Translational Medicine Research Center, Guizhou Medical University, Guizhou, China; c Dalian Institute of Chemical Physics, Chinese Academy of Sciences, Dalian, China; d The Second Hospital of Dalian Medical University, Dalian, China

**Keywords:** Triterpenoids, pancreatic lipase, human carboxylesterase 1, dual inhibitors, adipocyte adipogenesis

## Abstract

Pancreatic lipase (PL) is a well-known key target for the prevention and treatment of obesity. Human carboxylesterase 1A (hCES1A) has become an important target for the treatment of hyperlipidaemia. Thus, the discovery of potent dual-target inhibitors based on PL and hCES1A hold great potential for the development of remedies for treating related metabolic diseases. In this study, a series of natural triterpenoids were collected and the inhibitory effects of these triterpenoids on PL and hCES1A were determined using fluorescence-based biochemical assays. It was found that oleanolic acid (OA) and ursolic acid (UA) have the excellent inhibitory effects against PL and hCES1A, and highly selectivity over hCES2A. Subsequently, a number of compounds based on the OA and UA skeletons were synthesised and evaluated. Structure–activity relationship (SAR) analysis of these compounds revealed that the acetyl group at the C-3 site of UA (compound **41**) was very essential for both PL and hCES1A inhibition, with IC_50_ of 0.75 µM and 0.014 µM, respectively. In addition, compound **39** with 2-enol and 3-ketal moiety of OA also has strong inhibitory effects against both PL and hCES1A, with IC_50_ of 2.13 µM and 0.055 µM, respectively. Furthermore, compound **39** and **41** exhibited good selectivity over other human serine hydrolases including hCES2A, butyrylcholinesterase (BChE) and dipeptidyl peptidase IV (DPP-IV). Inhibitory kinetics and molecular docking studies demonstrated that both compounds **39** and **41** were effective mixed inhibitors of PL, while competitive inhibitors of hCES1A. Further investigations demonstrated that both compounds **39** and **41** could inhibit adipocyte adipogenesis induced by mouse preadipocytes. Collectively, we found two triterpenoid derivatives with strong inhibitory ability on both PL and hCES1A, which can be served as promising lead compounds for the development of more potent dual-target inhibitors targeting on PL and hCES1A.

## 1. Introduction

The morbidity and mortality of metabolic diseases such as hyperlipidaemia and diabetes are increasing year by year in developed and developing countries, which is inseparable from the changes in modern lifestyles and the increase in consumption of high-sugar and high-fat diets^1–3^. Studies have shown that elevated levels of fatty acids, cholesterol and other esters are important risk factors for metabolic diseases such as hypertension, arteriosclerosis, non-alcoholic fat, and type II diabetes^4^^,^^5^. Therefore, around the key targets of lipids, effective drugs that regulate lipid metabolism are an important direction for treating metabolic diseases and revealing the mechanism of metabolic diseases.

Mammalian carboxyesterase (CEs) is an important phase I metabolic enzyme, which is related to the metabolism or detoxification of endogenous substances, clinical drugs and environmental toxicants, and participates in a large number of ester drugs and other biotransformation and metabolic clearance of non-drug exogenous ester compounds^6–10^. Human carboxylesterase 1A (hCES1A) and human carboxylesterase 2A (hCES2A) are the two main subtypes in the human body. Although the amino acid sequences of hCES1A and hCES2A share 47% homology, there are significant differences in tissue distribution and substrate selectivity^9^^,^^11–13^. hCES1A is highly expressed in the liver, but relatively low in intestine and kidney. The preferred substrates of hCES1A are those compounds with relatively larger acyl groups and smaller alcohol structures, such as clopidogrel and oseltamivir^14^^,^^15^. In contrast, hCES2A is mainly distributed in the gastrointestinal tract, especially in the small intestine. Compounds containing smaller acyl groups and larger alcohol groups tend to be hydrolysed by hCES2A, such as irinotecan, capecitabine and flutamide^16^^,^^17^.

As an important serine hydrolases with the abundant distribution in the human hepatocytes and adipocytes, hCES1A plays a critical role in the hydrolysis of a large number of endogenous esters such as triglycerides and cholesteryl esters, so as to participate in physiological and pathological processes, such as cholesterol homeostasis, lipid metabolism and fatty liver^18^^,^^19^. Studies have shown that knocking out mice’s carboxylesterase 3 (Ces3, homologous to human hCES1A) will cause a significant decrease in plasma triglyceride and apolipoprotein B levels^20^. Meanwhile, the deficiency of Ces3 nullified the browning effect in white adipocytes, along with reduced adipogenesis in 3T3-L1 adipocytes^21^. In addition, a number of studies have shown that hCES1A expression is positively correlated with obesity, and its expression is up-regulated in fat cells in obese and type 2 diabetes patients^18^^,^^22^^,^^23^. Due to the key roles of hCES1A responsible for the enzymatic cleaving of triglyceride stores in hepatocytes, it has become an important target for the treatment of hypertriglyceridaemia^24–26^.

As the key enzyme of triglyceride hydrolysis in the intestine, pancreatic lipase (PL) catalyses the hydrolysis of the ester bond of triacylglycerols to monoacylglycerols and fatty acids, and contributes to 50–70% hydrolysis of total dietary fats^27^^,^^28^. Inhibition of PL activity could restrain the hydrolysis of dietary glycerides in food, so as to reduce the subsequent absorption of free fatty acids and monoacylglycerols. Therefore, PL has become a promising target for the adjuvant treatment of obesity and hypertriglyceridaemia^29^^,^^30^. In addition, inhibiting the activity of hCES1A could display multiple beneficial effects in both lipid and glucose homeostasis in genetic and diet-induced mouse models of obesity, insulin resistance and type 2 diabetes^18^. Thus, the discovery of potent dual-target inhibitors based on hCES1A and PL hold great potential for the development of remedies for treating related metabolic diseases such as hypertriglyceridaemia and obesity. However, the development of dual target inhibitors of hCES1A and PL is still in the blank stage.

To date, pharmaceutical chemists have found most CES inhibitors with good inhibitory activity. However, most of them are identified as potent and selective inhibitors against hCES2A. hCES1A inhibitors with high potency and selectivity are rarely reported, and their inhibitory activities to PL are not investigated^31–33^. At present, only GR148672x, a hCES1A inhibitor developed by GlaxoSmithKline, has entered the preclinical research stage, but its subtype selectivity data has not been disclosed^26^. Orlistat, a PL inhibitor, developed by Roche for the treatment of obesity and marketed as a prescription drug in New Zealand in 1998^34^. At present, orlistat remains the only PL inhibitor approved by the Food and Drug Administration (FDA) for obesity management. However, due to the non-negligible adverse effects, including oil stool, diarrhoea, fatsoluble vitamin deficiencies and hepatotoxicity, orlistat's application has been limited^35^^,^^36^. Thus, it is highly desirable to find potent dual inhibitors targeting hCES1A and PL for the prevention and treatment of related metabolic diseases.

Triterpenoids, structurally diverse natural products, are widely distributed in in various parts of plant including seeds, roots, flowers, leaves and fruits^37^^,^^38^. In the past decade, triterpenoids have been used as an effective structural template to find more effective lead compounds with a variety of pharmacological properties^39^, such as anti-tumour^40^, anti-virus^41^, anti-diabetes^42^, kidney-protective activity^43^, etc.

In this study, a series of triterpenoids were collected and the inhibitory effects of these triterpenoids on PL were assayed using 4-methylumbelliferyl oleate (4-MUO) as substrate probe^44^. After preliminary screening, we found that nine triterpenoids displayed good inhibitory effects against PL. More in-depth researches on the inhibitory effects of these nine triterpenoids against CES were assayed using *N*-alkylated d-luciferin methyl ester (NLMe) and fluorescent diacetate (FD) as specific optical substrate for hCES1A, and hCES2A, respectively. It was found that the ursolic acid (UA) and oleanolic acid (OA) have an excellent inhibitory effect on hCES1A and highly selectivity over hCES2A. Thus, we select UA and OA as the scaffolds and focus on their structural modifications to design and synthesise a batch of compounds to obtain potent dual target inhibitors of hCES1A and PL.

## 2. Experimental

### 2.1. Chemicals and reagents

Oleanolic acid, maslinic acid, hederagenin, ursolic acid, corosolic acid, asiatic acid, β-boswellic acid, glycyrrhetic acid, celastrol, betulin, betulinicaldehyde, betulinic acid, pachymic acid, ganoderic acid B, polygalacic acid, glycyrrhizic acid, lupeol, ginsenosideol F1, ginsenoside Rg1, ginsenoside Rg2, were purchased from Dalian Meilun Company (Beijing, China), ginsenoside Rd, ginsenoside R1, Notoginsenoside R1 were purchased from Sichuan Weikeqi Biotechnology Co., Ltd. (Chengdu, China), ginsenoside Re, ginsenoside Rh1 were purchased from Chengdu Pfeid Biotech Technology Co., Ltd. (Chengdu, China), ginsenoside Ro, ginsenoside Rh4, and ginsenoside F4 were purchased from Chenguang Bio (Handan, China). Fluorescent diacetate (FD, a fluorescent substrate for hCES2A) was purchased from TCI (Tokyo, Japan). Pancreatic lipase (PL, type II, Lot.SLBN9099V; EC 3.1.1.3), 4-methylumbelliferone (4-MU) were purchased from Sigma Aldrich (St. Louis, MO). 4-Methylumbelliferyl oleate (4-MUO) was obtained from J&K chemical (Beijing, China) as PL fluorescent substrate. NLMe was independently developed and synthesised by the laboratory, stored at −20 °C refrigerator [45]. Luciferin detection reagent (LDR) was purchased from Promega Biotech (Madison, WI). The pooled human liver microsomes from 50 donors (HLM, lot No. X008067) were purchased from Bioreclamation IVT (Baltimore, MD). 0.1 M McIlvane buffer (0.1 M citrate-Na_2_HPO_4_, pH 7.4) and 0.1 M phosphate buffered saline (PBS, pH 6.5 and 7.4) were prepared by using Milli-Q Water (Millipore, Bed-ford, CA). Bis-p-nitrophenyl phosphate (BNPP) was purchased from TCI (Tokyo, Japan). LC grade dimethyl sulfoxide (DMSO, Tedia, Fairfield, OH) was used as the stock solution of the compound and then stored at 4 °C until use. The stock solution of enzyme substrate (100 mM) was dissolved in dimethyl sulfoxide and stored at −20 °C. ^1^H NMR and ^13^C NMR spectra were recorded using Bruker Avance II (600 MHz) spectrometer with chemical shifts reported as ppm (in DMSO-d6 or CDCl_3_, TMS as an internal standard). High-resolution MS data were recorded with the 5600 Triple TOF quadrupole–time-of-flight mass spectrometer.

### 2.2. Synthesis of OA and UA derivatives

See the Supporting Information for more details.

Compound **30**, white solid, ^1^H NMR (600 MHz, DMSO) *δ* 12.05 (s, 1H), 5.19 (t, *J* = 3.4 Hz, 1H), 2.76 (dd, *J* = 13.8, 4.1 Hz, 1H), 2.55–2.46 (m, 1H), 2.32–2.27 (m, 1H), 2.00–1.81 (m, 3H), 1.77–1.75 (, 1H), 1.72–1.56 (m, 4H), 1.53–1.37 (m, 6H), 1.35–1.23 (m, 3H), 1.50–1.11 (m, 4H), 1.09–1.01 (m, 2H), 1.01–0.93 (t, 9H), 0.86 (d, 6H), 0.77 (s, 3H). ^13^C NMR (151 MHz, DMSO) *δ* 216.66, 179.05, 144.29, 121.88, 54.75, 47.09, 46.61, 46.11, 45.96, 41.93, 41.37, 39.29, 38.86, 34.12, 33.79, 33.28, 32.53, 32.29, 30.87, 27.69, 26.75, 25.94, 23.82, 23.45, 23.10, 21.58, 19.61, 17.16, 15.16. LC/MS (ESI): Calcd. for C_30_H_45_O_3_^−^ ([M-H]^−^) 453.3, Found. 453.3.

Compound **31**, white solid, ^1^H NMR (600 MHz, DMSO) *δ* 11.91 (s, 1H), 5.10 (s, 1H), 4.33 (m, 1H), 2.72–2.61 (m, 1H), 1.93 (s, 3H), 1.92–1.80 (m, 1H), 1.76–1.74 (m, 2H), 1.67–1.33 (m, 12H), 1.33–1.23 (m, 2H), 1.21–1.08 (m, 2H), 1.04 (s, 3H), 1.02–0.88 (m, 3H), 0.83–0.81 (m, 9H), 0.75 (s, 6H), 0.66 (s, 3H). ^13^C NMR (151 MHz, DMSO) *δ* 179.05, 170.61, 144.32, 121.87, 80.38, 54.99, 47.72, 47.31, 46.14, 45.92, 41.81, 41.27, 38.00, 37.71, 36.96, 33.77, 33.28, 33.02, 32.69, 32.54, 31.44, 30.86, 30.75, 28.23, 27.67, 26.01, 25.80, 23.84, 23.66, 23.35, 23.07, 22.68, 21.44, 18.25, 17.28, 17.10, 15.52. LC/MS (ESI): Calcd. for C_32_H_49_O_4_^−^ ([M-H]^−^) 497.3, Found. 497.3.

Compound **32**, white solid, ^1^H NMR (600 MHz, DMSO) *δ* 6.77 (d, *J* = 13.4 Hz, 2H), 5.21 (s, 1H), 4.39 (dd, *J* = 11.7, 4.4 Hz, 1H), 2.74 (dd, *J* = 13.3, 3.6 Hz, 1H), 2.00 (s, 3H), 1.89–1.86 (m, 1H), 1.81 (dd, *J* = 8.6, 3.0 Hz, 2H), 1.72 − 1.35 (m, 12H), 1.35–1.17 (m, 3H), 1.10 (s, 3H), 1.09–0.91 (m, 4H), 0.87 (t, 9H), 0.81 (d, 6H), 0.72 (s, 3H). ^13^C NMR (151 MHz, DMSO) *δ* 179.26, 170.60, 144.72, 121.63, 80.39, 55.01, 47.34, 46.60, 45.66, 41.77, 40.98, 39.31, 38.01, 37.71, 36.96, 34.13, 33.40, 33.08, 32.74, 30.91, 28.24, 27.49, 26.05, 23.99, 23.67, 23.35, 22.84, 21.44, 18.27, 17.37, 17.10, 15.53. LC/MS (ESI): Calcd. For C_32_H_52_NO_3_^+^ ([M + H]^+^) 498.4, Found. 498.4.

Compound **33**, white solid, ^1^H NMR (600 MHz, DMSO) *δ* 12.13 (bs, 1H), 5.16 (t, *J* = 3.3 Hz, 1H), 4.41 (dd, *J* = 11.7, 4.5 Hz, 1H), 2.74 (dd, *J* = 13.8, 4.1 Hz, 1H), 2.50–2.45 (m, 4H), 2.42 (s, 3H), 1.94–1.89 (m, 1H), 1.83–1.81 (m, 2H), 1.70–1.42 (m, 1H), 1.38–1.13 (m, 4H), 1.11 (s, 3H), 1.09–0.96 (m, 3H), 0.88 (d, 9H), 0.81 (s, 6H), 0.72 (s, 3H).^13^C NMR (151 MHz, DMSO) *δ* 179.04, 174.05, 173.87, 172.09, 144.33, 121.88, 80.47, 55.01, 47.29, 46.13, 45.92, 41.81, 41.27, 39.57, 39.33, 37.95, 37.79, 36.95, 33.77, 33.28, 32.69, 32.55, 30.86, 29.65, 29.25, 28.17, 27.67, 26.03, 23.84, 23.61, 23.35, 23.07, 18.25, 17.28, 17.08, 15.50. LC/MS (ESI): Calcd. For C_34_H_51_O_6_^−^ ([M-H]^−^) 555.3, Found. 555.3.

Compound **34**, white solid, ^1^H NMR (600 MHz, CDCl3) *δ* 5.28 (t, J = 3.4 Hz, 1H), 4.52–4.49 (m, 1H), 4.50 (dd, *J* = 9.8, 6.1 Hz, 1H), 2.83–2.80 (m, 1H), 2.32–2.24 (m, 2H), 2.01–1.96 (m, 1H), 1.93–1.84 (m, 2H), 1.81–1.69 (m, 2H), 1.69–1.49 (m, 11H), 1.47–1.40 (m, 2H), 2.06–0.60 (m, 53H), 1.36–1.27 (m, 2H), 1.24–1.15 (m, 2H), 1.14 (s, 3H), 1.09–1.04 (m 2H), 0.98–0.92 (m, 9H), 0.90 (s, 3H), 0.86 (d, 6H), 0.75 (s, 3H). ^13^C NMR (151 MHz, CDCl_3_) *δ* 183.46, 173.53, 143.59, 122.58, 80.56, 55.30, 47.54, 46.53, 45.84, 41.57, 40.95, 39.28, 38.06, 37.73, 36.99, 36.77, 33.79, 33.05, 32.54, 32.43, 30.67, 28.05, 27.67, 25.90, 23.57, 23.40, 22.90, 18.65, 18.17, 17.16, 16.71, 15.37, 13.73. LC/MS (ESI): Calcd. for C_34_H_53_O_4_^−^ ([M-H]^−^) 525.4, Found. 525.4.

Compound **35**, white solid, ^1^H NMR (600 MHz, CDCl_3_) *δ* 5.27 (t, *J* = 3.4 Hz, 1H), 4.51–4.49 (m, 1H), 2.83–2.80 (m, 1H), 2.35–2.24 (m, 2H), 2.01–1.96 (m, 1H), 1.94–1.84 (m, 2H), 1.80–1.68 (m, 2H), 1.68–1.52 (m, 10H), 1.51–1.38 (m, 2H), 1.38–1.23 (m, 7H), 1.23–1.11 (m, 5H), 1.10–1.02 (m, 2H), 0.96–0.88 (m, 12H), 0.86 (d, *J* = 5.4 Hz, 6H), 0.75 (s, 3H). ^13^C NMR (151 MHz, CDCl_3_) *δ* 183.77, 173.71, 143.59, 122.58, 80.56, 55.29, 47.54, 46.54, 45.83, 41.56, 40.93, 39.28, 38.05, 37.73, 36.99, 34.82, 33.78, 33.06, 32.53, 32.43, 31.35, 30.67, 28.05, 27.66, 25.91, 24.84, 23.57, 23.56, 23.39, 22.89, 22.32, 18.17, 17.16, 16.72, 15.37, 13.92. LC/MS (ESI): Calcd. for C_36_H_57_O_4_^−^ ([M-H]^−^) 553.4, Found. 553.4.

Compound **36**, white solid, ^1^H NMR (600 MHz, DMSO) *δ* 5.18 (s, 1H), 4.29 (d, *J* = 5.2 Hz, 1H), 3.54 (s, 3H), 3.01–2.97 (m, 1H), 2.79–2.76 (m, 1H), 2.05–1.91 (m, 1H), 1.89–1.72 (m, 2H), 1.65–1.56 (m, 2H), 1.55–1.28 (m, 11H), 1.25–1.12 (m, 2H), 1.08 (m, 3H), 1.08–0.90 (m, 3H), 0.88 (d, 9H), 0.85 (s, 3H), 0.68 (s, 3H), 0.65 (s, 3H). ^13^C NMR (151 MHz, DMSO) *δ* 177.62, 143.92, 122.32, 77.27, 55.23, 51.90, 47.50, 46.51, 45.86, 41.66, 41.36, 39.27, 38.84, 38.50, 37.03, 33.61, 33.21, 32.74, 32.46, 30.82, 28.67, 27.62, 27.40, 26.12, 23.82, 23.36, 23.06, 18.44, 17.02, 16.48, 15.53. LC/MS (ESI): Calcd. for C_31_H_51_O_3_^+^ ([M + H]^+^) 471.4, Found. 471.4.

Compound **37**, white solid, ^1^H NMR (600 MHz, CDCl_3_) *δ* 5.19 (t, *J* = 3.5 Hz, 1H), 3.55 (d, *J* = 10.9 Hz, 1H), 3.22 (dd, *J* = 11.2, 4.0 Hz, 2H), 1.98 (dd, *J* = 13.6, 4.2 Hz, 1H), 1.90–1.85 (m, 3H), 1.77–1.69 (m, 2H), 1.65–1.53 (m, 6H), 1.51–1.39 (m, 7H), 1.36–1.28 (m, 3H), 1.17 (s, 3H), 1.08–1.05 (m, 1H), 1.00 (s, 3H), 0.99–0.96 (m, 2H), 0.94 (d, 6H), 0.88 (d, 6H), 0.79 (s, 3H), 0.77–0.72 (m, 1H). ^13^C NMR (151 MHz, CDCl_3_) *δ* 144.21, 122.37, 79.00, 69.71, 55.16, 47.57, 46.46, 42.34, 41.72, 39.78, 38.78, 38.59, 36.94, 36.93, 34.08, 33.20, 32.57, 31.03, 30.96, 28.09, 27.22, 25.95, 25.55, 23.58, 23.53, 22.00, 18.35, 16.73, 15.58, 15.52. LC/MS (ESI): Calcd. for C_30_H_51_O_2_^+^ ([M + H]^+^) 443.3, Found.443.3.

Compound **38**, white solid, ^1^H NMR (600 MHz, DMSO) *δ* 6.77 (d, *J* = 12.1 Hz, 2H), 5.21 (s, 1H), 4.28 (d, *J* = 5.1 Hz, 1H), 3.01–2.98 (m, 1H), 2.73 (dd, *J* = 13.3, 3.6 Hz, 1H), 1.88 (td, *J* = 13.5, 3.3 Hz, 1H), 1.80 (dd, *J* = 8.6, 3.0 Hz, 2H), 1.63 (t, *J* = 13.5 Hz, 2H), 1.57–1.37 (m, 9H), 1.36–1.26 (m, 2H), 1.23–1.21 (m, 1H), 1.11–1.09 (m, 4H), 1.04 (d, *J* = 11.4 Hz, 1H), 0.93–0.91 (m, 1H), 0.87 (q, 12H), 0.72 (s, 3H), 0.68–0.66 (m, 4H). ^13^C NMR (151 MHz, DMSO) *δ* 179.26, 144.69, 121.75, 77.29, 55.28, 47.58, 46.61, 45.66, 41.74, 40.97, 39.32, 38.85, 38.52, 37.07, 34.13, 33.42, 33.09, 32.92, 30.91, 28.71, 27.49, 27.43, 26.11, 23.99, 23.37, 22.84, 18.49, 17.41, 16.49, 15.58. LC/MS (ESI): Calcd. for C_30_H_50_NO_2_^+^ ([M + H]^+^) 456.4, Found. 456.4.

Compound **39**, light yellow solid, ^1^H NMR (600 MHz, CDCl_3_) *δ* 6.35 (s, 1H), 5.95 (s, 1H), 5.34 (t, *J* = 3.5 Hz, 1H), 2.88–2.83 (m, 1H), 2.15–2.06 (m, 2H), 2.04–1.97 (m, 2H), 1.92–1.88 (m, 1H), 1.81–1.69 (m, 4H), 1.65–1.60 (m, 4H), 1.54–1.50 (m, 2H), 1.39–1.33 (m, 4H), 1.22 (d, 6H), 1.14 (s, 3H), 1.11 (s, 3H), 0.94 (s, 3H), 0.91 (s, 3H), 0.83 (s, 3H). ^13^C NMR (151 MHz, CDCl_3_) *δ* 201.06, 183.33, 143.87, 143.72, 128.19, 122.08, 53.83, 46.58, 45.59, 43.89, 43.03, 41.99, 41.15, 39.98, 38.45, 33.78, 33.05, 32.44, 32.37, 31.59, 30.68, 27.58, 27.18, 26.97, 25.87, 23.54, 23.38, 22.82, 22.66, 21.80, 19.67, 18.68, 17.44, 14.12. LC/MS (ESI): Calcd. for C_30_H_43_O_4_^−^ ([M-H]^−^) 467.3, Found. 467.3.

Compound **40**, white solid, ^1^H NMR (600 MHz, DMSO) *δ* 11.95 (s, 1H), 5.16 (t, *J* = 3.3 Hz, 1H), 2.55–2.45 (m, 1H), 2.32–2.88 (m, 1H), 2.12 (d, *J* = 11.3 Hz, 1H), 1.96–1.89 (m, 3H), 1.85–1.73 (m, 2H), 1.64–1.36 (m, 9H), 1.36–1.22 (m, 4H), 1.06 (s, 3H), 1.04–1.02 (m, 1H), 1.00–0.92 (q, 12H), 0.85–0.76 (d, 6H). ^13^C NMR (151 MHz, DMSO) *δ* 216.70, 178.75, 138.71, 124.95, 54.68, 52.93, 47.36, 47.06, 46.49, 42.26, 38.96, 36.77, 36.64, 34.16, 32.57, 30.66, 28.02, 26.84, 24.28, 23.64, 23.46, 21.62, 21.52, 19.60, 17.49, 17.28, 15.31. LC/MS (ESI): Calcd. for C_30_H_45_O_3_^−^ ([M-H]^−^) 453.3, Found. 453.3.

Compound **41**, white solid, ^1^H NMR (600 MHz, DMSO) *δ* 11.95 (s, 1H), 5.14 (t, *J* = 3.4 Hz, 1H), 4.43–4.40 (m, 1H), 2.12 (d, *J* = 11.3 Hz, 1H), 2.01 (s, 3H), 1.98–1.92 (m, 1H), 1.91–1.77 (m, 3H), 1.67–1.41 (m, 10H), 1.39–1.22 (m, 4H), 1.07 (s, 3H), 1.04–1.00 (m, 3H), 0.92 (d, 6H), 0.86–0.84 (m, 1H), 0.85–0.80 (m, 9H), 0.77 (s, 3H). ^13^C NMR (151 MHz, DMSO) *δ* 178.75, 170.58, 138.71, 124.91, 80.37, 54.97, 52.83, 47.29, 47.24, 42.13, 38.96, 38.88, 38.17, 37.72, 36.89, 36.78, 32.97, 30.64, 28.27, 28.00, 24.26, 23.70, 23.29, 21.54, 21.45, 18.24, 17.52, 17.35, 17.16, 15.63. LC/MS (ESI): Calcd. for C_32_H49O_4_^−^ ([M-H]^−^) 497.3, Found. 497.3.

Compound **42**, white solid, ^1^H NMR (600 MHz, DMSO) *δ* 12.14 (ds, 3H), 5.13 (s, 1H), 4.41 (dd, *J* = 11.5, 4.4 Hz, 1H), 2.48–2.41 (m, 4H), 2.11 (d, *J* = 11.4 Hz, 1H), 1.94–1.80 (m, 4H), 1.66–1.38 (m, 11H), 1.37–1.21 (m, 5H), 1.05 (s, 3H), 1.02–1.96 (m, 2H), 0.91 (d, 6H), 0.82 (d, 9H), 0.76 (s, 3H). ^13^C NMR (151 MHz, DMSO) *δ* 178.75, 174.12, 173.87, 172.09, 138.71, 124.91, 80.47, 54.99, 52.83, 47.29, 47.22, 38.96, 38.88, 38.12, 37.80, 36.88, 36.78, 32.96, 30.63, 29.64, 29.42, 29.25, 28.21, 28.01, 24.26, 23.71, 23.65, 23.30, 21.54, 18.22, 17.51, 17.35, 17.14, 15.61. LC/MS (ESI): Calcd. for C_32_H_51_O_6_^−^ ([M-H]^−^) 555.3, Found. 555.3.

Compound **43**, light yellow solid. ^1^H NMR (600 MHz, CDCl_3_) *δ* 6.37 (s, 1H), 5.94 (s, 1H), 5.31 (t, *J* = 3.4 Hz, 1H), 2.22 (d, *J* = 11.3 Hz, 1H), 2.19–2.12 (m, 1H), 2.10–2.05 (m, 1H), 2.04–1.99 (m, 1H), 1.91–1.83 (m, 2H), 1.76–1.66 (m, 4H), 1.63–1.48 (m, 6H), 1.42–1.39(m, 1H), 1.36–1.29 (m, 3H), 1.24 (s, 3H), 1.22 (s, 3H), 1.11 (s, 3H), 1.09 (s, 3H), 0.96–0.95 (m, 3H), 0.87 (s, 3H), 0.85 (s, 3H). ^13^C NMR (151 MHz, CDCl_3_) *δ* 201.08, 183.29, 143.73, 138.40, 128.34, 125.20, 53.85, 52.66, 48.02, 43.89, 42.94, 42.38, 40.17, 38.92, 38.79, 38.31, 36.62, 32.76, 30.57, 27.90, 27.23, 26.92, 23.99, 23.51, 23.30, 21.78, 21.13, 19.77, 18.67, 17.51, 16.94. LC/MS (ESI): Calcd. for C_30_H_43_O_4_^−^ ([M-H]^−^) 467.3, Found. 467.3.

### 2.3. Florescence-based enzyme inhibition assays

#### 2.3.1. Inhibition of triterpenoids on PL-mediated 4-MUO hydrolysis

4-MUO was used as a fluorescent probe substrate to detect the inhibitory effect of triterpenoids on PL in a black standard 96-well plate, while DMSO and orlistat were used as negative and positive controls, respectively. In addition, the background fluorescence in the absence of an enzyme source (PL) (add the same amount of citrate-Na_2_HPO_4_ buffer) was measured. The detailed method has been reported in our previous articles^46^^,^^47^, the total volume of the incubation mixture was 200 µL, in short, the compound and PL enzyme source were placed in 0.1 M citrate-Na_2_HPO_4_ buffer and pre-incubated at 37 °C for 3 min. Then start the reaction by adding 2 µL of 4-MUO probe (3 µM, final concentration). The fluorescence signal of 4-MU (hydrolytic metabolite) was detected by multi-mode microplate reader (SpectraMax M4, Molecular Devices, Urstein, Austria) with continuous oscillation for 20 min and recorded and analysed at 60 s intervals. The excitation wavelength and emission wavelength of 4-MU were set at 340 nm and 460 nm, respectively, and the gain value was 500. The inhibitors are all prepared with DMSO, the final DMSO concentration was 1% (v/v, does not affect the catalytic activity), and the remaining activity was calculated according to the following formula: residual activity (%) = (inhibitor-hydrolysate fluorescence intensity under background fluorescence)/(the hydrolysate fluorescence intensity of the background fluorescence of the negative control (DMSO only)×100%.

#### 2.3.2. Inhibition of triterpenoids on hCES1A-mediated NLMe hydrolysis

*N*-Alkylated d-luciferin methyl ester (NLMe), a Specific probe for hCES1A^45^, was used for assessing the inhibitory effects for triterpenoids, and BNPP was used as a positive inhibitor of hCES1A. The system for the determination of hCES1A was as follows: first, each compound (2 µL) and HLM (5 µL, 0.02 mg/mL, initial concentration) were incubated in PBS (91 µL, pH 6.5) for 3 min at 37 °C. Then NLMe (2 µL, 0.15 mM) was added to start the reaction. After shaking the reaction in 37 °C incubator for 10 min, 50 µL of reaction solution was removed and mixed with LDR (50 µL) to terminate the reaction. A multi-mode microplate reader was used to detect the luminescence signal of NL (the hydrolysed metabolite of NLMe). The luminescence product of NL was measured at 580 nm (gain: 140). The final volume ratio of DMSO was less than 1% (V/V).

#### 2.3.3. Inhibition of triterpenoids on hCES2A-mediated FD hydrolysis

Fluorescent diacetate (FD) was used as the hCES2A specific probe substrate to assay the inhibitory effect of natural triterpenoids on hCES2A. The entire detailed operation process refers to the previously reported method^32^, and finally the FD mixture was put into the multi-mode microplate reader for analysis and detection. The excitation wavelength of the hydrolysed metabolites of FD was 480 nm and the emission wavelength was 525 nm.

#### 2.3.4. Inhibition of triterpenoids on DPP-IV-mediated GP-BAN hydrolysis

Glycyl-prolyl-*N*-butyl-4-amino-1,8-naphthalimide (GP-BAN) was used as a substrate to evaluate the inhibitory effects of triterpenoids against dipeptidyl peptidase-IV (DPP-IV)^48^. The details for DPP-IV inhibition has been reported previously^49^^,^^50^. The fluorescent signal of the hydrolytic product (BAN) was measured by a fluorescence microplate reader at an excitation wavelength of 430 nm, an emission wavelength of 535 nm (gain = 500).

#### 2.3.5. Inhibition of triterpenoids on BuChE-mediated BTCH hydrolysis

To assay the inhibitory effects of triterpenoids on Butyrylcholinesterase (BuChE), butyrylthiocholine (BTCH) was used as a substrate for BuChE, The total volume of the system was 100 µL. Including 2 µL DMSO/inhibitor, 2 µL human serum, 84 µL buffered PBS (pH 7.4, 0.1 M), 10 µL substrate iodised butyrylthiocholine (0.3 mM, final concentration) and 2 µL developer 5–5′-dithiobis(2-nitrobenzoic acid) (DTNB) (1 mM, final concentration). The assay protocol of trypsin inhibition was depicted previously^32^. All measurements were performed in triplicates.

### 2.4. Inhibition kinetic analysis

The IC_50_ (the concentration of the inhibitor that reduces the enzyme activity by 50%) of the compound with strong inhibitory ability (compound 3**9**/**41**) against PL and hCES1A was assayed, using the above inhibitor detection conditions. Subsequently, various concentrations of substrates (selected according to *K_m_*) and different concentrations of inhibitors were used to determine the corresponding reaction rate, and the second slope graph of the Lineweaver–Burk diagram was used as a function of the inhibitor to calculate the corresponding inhibition constant (*K_i_*) value. All kinetic data were fitted by the following kinetic equations (a–c), for competitive (a), non-competitive (b) and mixed inhibition (c):

(a) $$V=\left(V_{\max}S\right)/\left[K_{m}\left(1+I/K_{i}\right)+S\right]$$

(b) $$V=\left(V_{\max}S\right)/\left[\left(K_{m}+S\right)\times \left(1+I/K_{i}\right)\right]$$

(c) $$V=\left(V_{\max}S\right)/\left[\left(K_{m}+S\right)\times \left(1+I/\alpha K_{i}\right)\right]$$

where *V* is the hydrolytic velocity of the reaction, *V*_max_ is the maximum velocity. *S* and *I* are substrate (4-MUO, NLMe) and inhibitor (compound **39**/**41**) concentrations, respectively. *K_i_* is the inhibition constant of the tested inhibitor against the target PL and hCES1A; *K_m_* is the Michaelis constant (the substrate concentration at half of the *V*_max_).

### 2.5. Statistical analysis

All measurements were made in triplicate and the data obtained in the experiment were shown as mean ± SD. The IC_50_ and *K_i_* values of the compounds with strong inhibitory effect were evaluated by nonlinear regression with graphpad prism 7.0 software (GraphPad Software, Inc., La Jolla, CA).

### 2.6. Molecular docking

To explore the binding mechanisms of compounds **39** and **41** against two proteins at the molecular level, the structures of PL (PDB: 1ETH) and hCES1A (PDB: 1MX5) were download from https://www.rcsb.org/ as receptors and compounds **39** and **41** as ligand, molecular docking was performed with AutoDock Vina (Version 1.1.2) based on Lamarckian genetic algorithm^51^. Hydrogen atoms were added followed by assigning the Kollman charges. The centre of grid box was set to 60 × 60 × 60 Å^3^ with the spacing of 0.375, enclosing the known binding sites reported previously^52^^,^^53^, i.e. the Ser221 of hCES1A and the Ser153 for active site (Site I) and the surface between lipase and colipase for interface site of PL (Site II). Favourable binding processes with the lowest binding energy were indicated for the follow-up calculation.

### 2.7. Cytotoxicity assays

The effect of compounds **39** and **41** on cell viability of the 3T3-L1cells was measured using Cell Counting Kit-8 (Dalian Meilun Biotechnology Co., Ltd., Dalian, China). Briefly, 3T3-L1 cells (5 × 10^4^/mL, 100 µL) were seeded in the 96-well plate, After 24 h incubation, then the cells were treated with different concentrations of compounds **39** and **41** (0–100 µM) for another 48 h. Then, CCK-8 (10%, v/v, 100 µL) was added to each well and incubated at 37 °C for additional 2 h. The absorbance was determined by microplate reader at 450 nm. The percentage of cell viability was calculated towards control. Each condition included replicate wells with at least four independent repeats.

### 2.8. Cell culture

3T3-L1 cells (ATCC CL-173, Manassas, VA) were cultured in Meilunbio Dulbecco's Modified Eagle Medium/F:12 (DMEM/F:12), supplemented with 10% FBS, and 1% penicillin–streptomycin at 37 °C with 5% CO_2_ The method of 3T3-L1 cell differentiation into adipocytes refers to our previous article^54^. The cells were planted in a well plate and cultured for 48 h after the cells were 100% confluent. Mix inhibitors compounds **39** and **41** with solution I (10 µg/mL insulin in DMEM/F: 12 medium, 0.5 mM 3-isobutyl-1-methylxanthine and 1 mM dexamethasone) to different concentrations, Then added to different wells and incubated for 48 h, then the inhibitor was mixed with solution II (5 µg/mL insulin in DMEM/F: 12 medium), then added to the wells of the same concentration and incubated for 48 h. Then, the medium in the well was replaced with DMEM/F:12 medium with different concentrations of inhibitors, and the culture was continued for 48 h.

### 2.9. Oil red O staining

The lipid droplets of 3T3-L1 cells were stained with Oil Red O stain kit (Beijing solebo Technology Co., Ltd., Beijing, China) after cultured for 48 h. In short, 3T3-L1 cells were washed with PBS 2 times and fixed with ORO fixative for 20–30 min, and then washed again with deionised water for 3 times. Then, 3T3-L1 cells were washed with 60% isopropanol for 5 min, immersed in oil red O solution for 10–20 min, and washed with deionised water for 3–5 times. Mayer's haematoxylin staining solution was added, the nucleus was stained for 1–2 min, then washed for 3–5 times, and Oro buffer solution was added for 1 min. Finally, distilled water was added to cover the cells. The stained lipid droplets were observed under inverted microscope (Lecai DMI8).

## 3. Results and discussion

### 3.1. Screening of PL inhibitors from natural triterpenoids

**Scheme 1.**
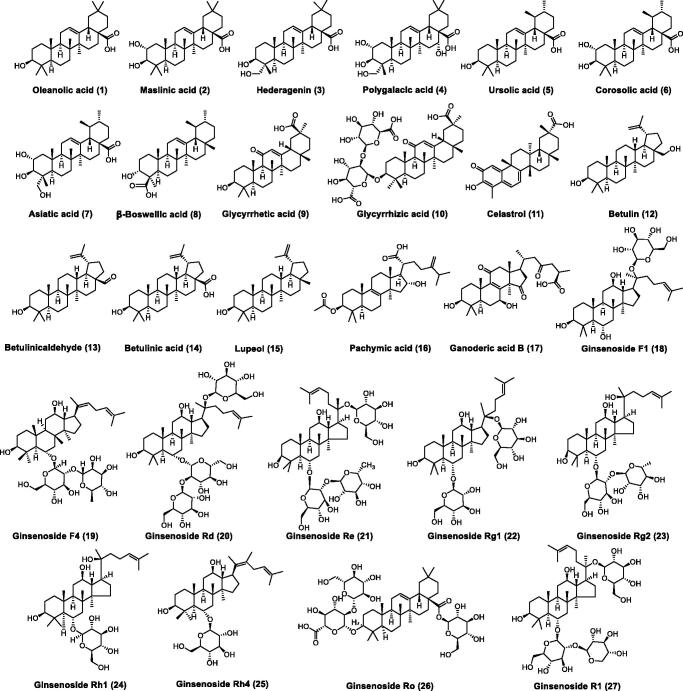
Chemical structure of natural triterpenoids.

In this study, the inhibitory effects of 27 natural triterpenoids (Scheme 1) against PL were determined. As shown in Table 1, oleanolic acid (**1**) and ursolic acid (**5**) were found to display good inhibitory effects on PL, while natural pentacyclic triterpenoids (**2–4, 6, 7**) with more hydroxyl group compared to oleanolic acid (**1**) and ursolic acid (**5**), displayed less potency towards PL. β-Boswellic acid (**8**) with carboxyl group at the C-23 site displayed potent inhibitory effects against PL, while glycyrrhetic acid (**9**) with carboxyl group at the C-30 site exhibited less inhibitory effects. Furthermore, glycyrrhizic acid (**10**) with the glycosidic group at the C-3 site demonstrated poor inhibitory effects on PL. Structure–activity relationships (SAR) analysis suggested that natural pentacyclic triterpenoids with more hydroxyl group and glycosidic group may be not beneficial for PL inhibition. Celastrol (**11**) and betulin (**12**) showed good inhibitory effects on PL, while betulin homologues (**13**–**15**) displayed a decrease of inhibitory effects on PL. In addition, pachymic acid (**16**), ganoderic acid B (**17**) and ginsenosides (**18**–**27**) exhibited less potent or poor inhibitory effects on PL, which suggested that the long alkyl chain at the C-20 site of triterpenoids and glycosidic group could be unbeneficial for PL inhibition.

**Table 1.** The inhibitory effects of natural triterpenes against PL

Compound	IC_50_ (µM) PL	Compound	IC_50_ (µM) PL
**1**	8.63 ± 0.84	**15**	>100
**2**	16.02 ± 4.59	**16**	>100
**3**	13.01 ± 2.03	**17**	>100
**4**	>100	**18**	>100
**5**	6.16 ± 0.97	**19**	74.46 ± 28.42
**6**	12.79 ± 1.44	**20**	29.72 ± 5.02
**7**	12.93 ± 1.87	**21**	>100
**8**	0.42 ± 0.05	**22**	>100
**9**	28.33 ± 9.47	**23**	>100
**10**	>100	**24**	>100
**11**	4.69 ± 0.37	**25**	>100
**12**	7.47 ± 1.68	**26**	>100
**13**	>100	**27**	>100
**14**	>100	**28**	0.2345 ± 0.029

Data were shown as mean ± SD (*n* = 3). 28* Orlistat, a PL positive inhibitors.

### 3.2. Inhibitory effects of natural triterpenoids against hCES1A and hCES2A

Above studies shown that there were nine natural triterpenoids with moderate inhibitory effect against PL (IC_50_< 20 µM). Thus, a further investigation was carried out to evaluate the inhibitory effects of these compounds on hCES1A and hCES2A using NLMe and FD as specific optical substrate, respectively. As shown in Table 2, except for β-boswellic acid (**8**) and betulin (**12**), other seven triterpenoids showed moderate to strong inhibitory effect on the hydrolysis of hCES1A-mediated NLMe. It was found from these natural triterpenoids that the OA (**1**) and UA (**5**) have the good inhibitory effects against hCES1A and PL, and highly selectivity over hCES2A. These results encouraged us to design and synthesise a batch of compounds based on the scaffolds of OA and UA to obtain potent dual target inhibitors of hCES1A and PL.

**Table 2.** The inhibitory effects of natural triterpenes against hCES1A, hCES2A and PL

Compound	IC_50_ (µM)	IC_50_ (µM)	IC_50_ (µM)
	hCES1A	hCES2A	PL
**1**	0.04 ± 0.003	6.02 ± 0.54	8.63 ± 0.84
**2**	0.12 ± 0.01	5.91 ± 1.65	16.02 ± 4.59
**3**	0.04 ± 0.004	9.22 ± 1.43	13.01 ± 2.03
**5**	0.04 ± 0.004	7.38 ± 1.10	6.16 ± 0.97
**6**	0.34 ± 0.04	14.45 ± 2.01	12.79 ± 1.44
**7**	0.17 ± 0.02	65.08 ± 6.41	12.93 ± 1.87
**8**	>100	/	0.42 ± 0.05
**11**	14.27 ± 3.03	2.31 ± 0.12	4.69 ± 0.37
**12**	>100	/	7.47 ± 1.68
**28**	0.035 ± 0.003	1.83 ± 0.26 (nM)	0.23 ± 0.03
**29**	0.36 ± 0.03	3.08 ± 0.77	>100

Data were shown as mean ± SD (*n* = 3). 28* Orlistat, a PL positive inhibitor. 29* BNPP, broad-spectrum inhibitor of hCES1A and hCES2A.

### 3.3. Synthesis of OA and UA derivatives

**Scheme 2.**
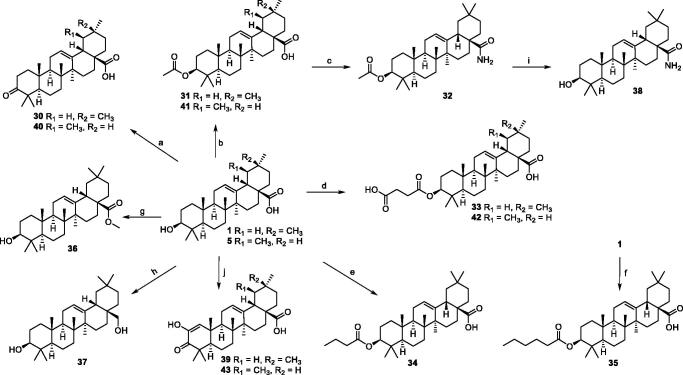
Synthetic route of OA and UA derivatives. Reagents and conditions: (a) Jones reagent, acetone, 0 °C, 1 h, 72–80%; (b) acetic anhydride, pyridine, rt, 24 h, 88–92%; (c) (COCl)_2_, CH_2_Cl_2_, rt, 2 h, then conc. ammonia, toluene, 4–8 °C, 2 h, 64%; (d) succinic anhydride, DMAP, CH_2_Cl_2_, rt, 24 h, 85–93%; (e) *n*-butyric anhydride, DMAP CH_2_Cl_2_, rt, 24 h, 64%; (f) *n*-hexanoic anhydride, DMAP, CH_2_Cl_2_, rt, 24 h, 71%; (g) CH_3_I, K_2_CO_3_, acetone, rt, 12 h, 88%; (h) LiALH_4_, THF, rt, 24 h, 60%; (i) NaOH, MeOH/THF, 40 °C, 5 h, 86%; (j) Jones reagent, acetone, 0 °C, 1 h, then, *t*-BuOK/*t*-BuOH, THF, 31–34%.

Compounds **31–43** were synthesised according to Scheme 2. Compounds **30** and **40** were obtained in high yield from OA (**1**) and UA (**5**) with the Jones’ reagent, respectively. OA (**1**) and UA (**5**) were introduced acetyl in C-3 with acetic anhydride in pyridine to obtain compounds **31** and **41** with high yield (88–92%), respectively. Compound **31** was then treated with oxalyl chloride, without isolation, further reacted with concentrated ammonia to afford compound **32** in a yield of 64%. The 3β-hydroxy group of OA (**1**) and UA (**5**) were reacted with succinic anhydride in the presence of 4-dimethylaminopyridine (DMAP) to obtain the target products **33** and **42,** respectively. OA (**1**) reacted with *n*-butyric anhydride and *n*-hexanoic anhydride to afford compounds **34** and **35,** respectively. Reaction of the iodomethane with OA furnished the target compound **36**. The OA was reduced with lithium aluminium hydride to afford the compound **37**. Compound **32** was hydrolysed under the effectiveness of NaOH to afford compound **38** in 86% yield. OA (**1**) and UA (**5**) were treated with Jones’ reagent, and further reacted with *t*-BuOK to afford compounds **39** and **43**, respectively.

### 3.4. Inhibitory effects of OA and UA derivatives against PL and hCES1A

A batch of OA and UA derivatives were synthesised. We further evaluated the inhibitory effects of these fourteen derivatives on PL and hCES1A. As shown in Table 3, the 3-keto-OA derivative (**30**) exhibited similar trends in PL inhibition as OA, while the compound **31** with carbonyl group at the C-3 site resulted in an increase of inhibitory effect on PL compared with OA. Compounds **32** and **38** exhibited relatively low inhibitory activities against PL as compared with compound **31** and OA, respectively, suggesting that the introduction of amides group at C-28 results in a loss of potency. Notably, replacement of the C-3 ethyl ester group with 3-O-β-carboxypropionyl, *n*-butyric and *n*-hexanoic in compounds **33**–**35** led to a dramatically decrease in the inhibitory effects against PL. These results suggested that the structural modifications on the C-3 hydroxyl group of OA with bigger acyl groups such as 3-O-β-carboxypropionyl, *n*-butyric and *n*-hexanoic were unbeneficial for the development of potent inhibitors against PL. Alcohols (**36**) and esters (**37**) derivatives displayed decrease inhibitory effects towards PL compared with OA, while compound **39** with 2-enol and 3-ketal moiety exhibited potent inhibitory effect on PL. Consistently, UA derivatives (**40**–**43**) exhibited similar trends in PL inhibition as OA derivatives (**30**, **31**, **34** and **39**). Further evaluate the inhibitory effects of fourteen derivatives on hCES1A showed that except for compounds **35** and **37**, other twelve derivatives showed moderate to strong inhibitory effect on the hydrolysis of hCES1A-mediated NLMe.

**Table 3.** The inhibitory effects of OA and UA derivatives against PL and hCES1A.

Compound	IC_50_ (µM) PL	IC_50_ (µM) hCES1A	Compound	IC_50_ (µM) PL	IC_50_ (µM) hCES1A
**30**	7.51 ± 1.08	0.047 ± 0.003	**37**	11.78 ± 1.60	4.106 ± 0.77
**31**	3.67 ± 0.72	0.051 ± 0.005	**38**	7.83 ± 1.21	0.48 ± 0.06
**32**	7.02 ± 0.59	0.11 ± 0.013	**39**	2.13 ± 0.19	0.055 ± 0.006
**33**	36.39 ± 5.17	0.043 ± 0.003	**40**	6.94 ± 1.37	0.93 ± 0.18
**34**	>100	0.24 ± 0.049	**41**	0.75 ± 0.12	0.014 ± 0.002
**35**	>100	1.82 ± 0.29	**42**	20.87 ± 1.78	0.028 ± 0.003
**36**	30.59 ± 3.58	0.856 ± 0.15	**43**	5.21 ± 1.24	0.032 ± 0.003

Data were shown as mean ± SD (*n* = 3).

### 3.5. Sar summary of triterpenoids

**Figure 1.**
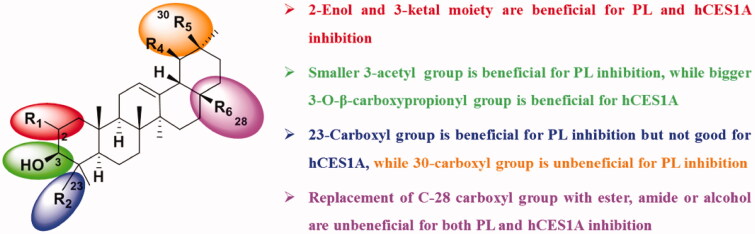
SAR summary of triterpenoids.

Based on these results of the inhibitory effects of nature triterpenoids and 14 derivatives on PL and hCES1A, the structure–PL/hCES1A inhibition relationships of these triterpenoids are summarised as follows, (1) natural pentacyclic triterpenoids with more hydroxyl group and glycosidic group are not beneficial for PL inhibition, (2) triterpenoids with 2- enol and 3-ketal moiety are beneficial for PL and hCES1A inhibition, (3) structural modifications on the C-3 hydroxyl group with bigger acyl groups were unbeneficial for PL inhibition, while replacement of C-3 hydroxyl group with ester led to improved inhibitory effects towards hCES1A, (4) long alkyl chain at the C-20 site of triterpenoids and glycosidic group could be unbeneficial for PL inhibition, (5) carboxyl group at the C-23 site is beneficial for PL inhibition but not good for hCES1A inhibition, while carboxyl group at the C-30 site is unbeneficial for PL inhibition, and (6) replacement of C-28 carboxyl group with ester, amide or alcohol are unbeneficial for both PL and hCES1A inhibition (Figure 1). These key findings are very helpful for pharmacochemists to better understand the SAR of triterpenoids for both PL and hCES1A inhibition and to develop novel dual inhibitors using triterpenoids as leading compounds.

### 3.6. Inhibition kinetic analyses

**Figure 2.**
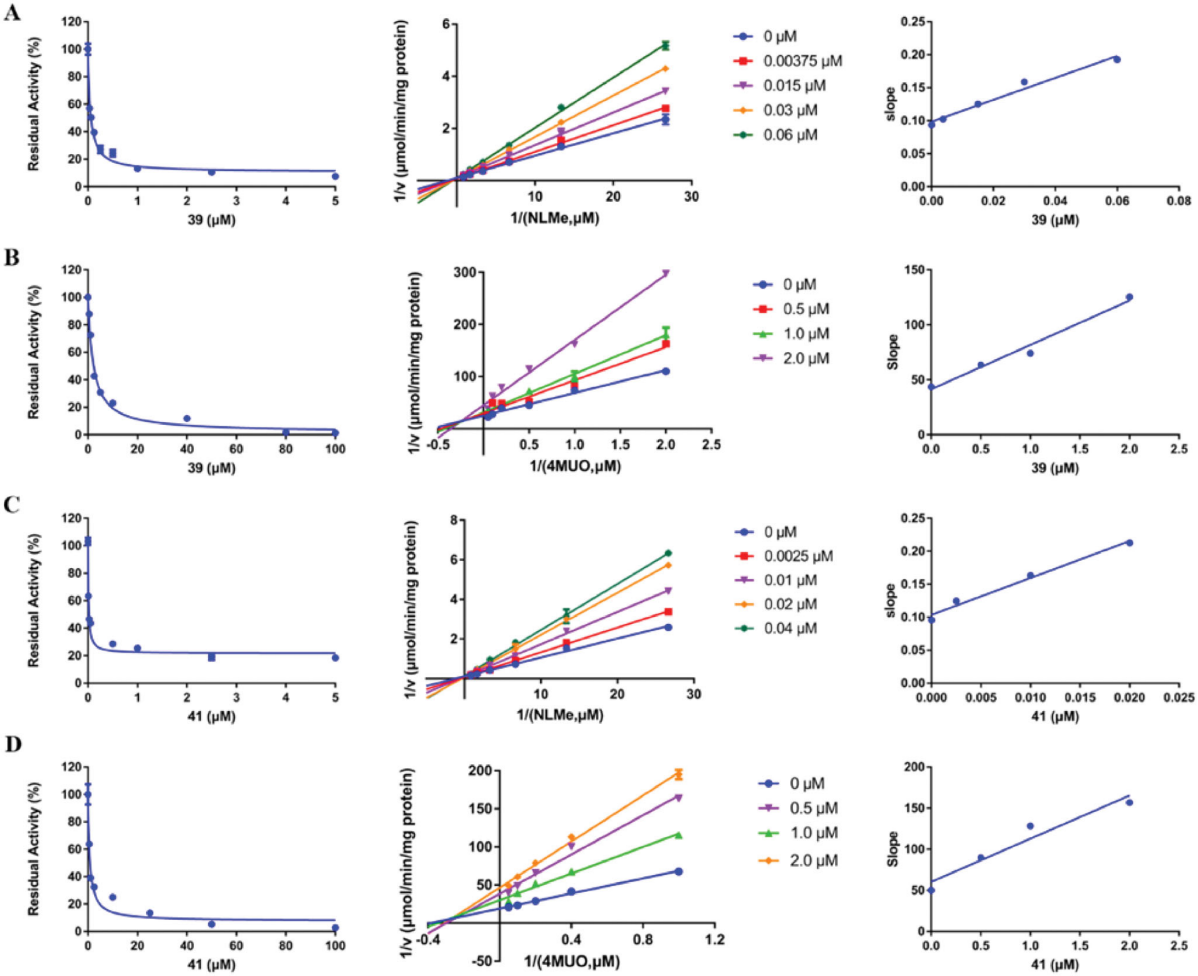
(A) Inhibition behaviours of **39** against hCES1A-mediated NLMe hydrolysis. (B) Inhibition behaviours of **39** against PL mediated 4-MUO hydrolysis. (C) Inhibition behaviours of **41** against hCES1A-mediated NLMe hydrolysis. (D) Inhibition behaviours of **41** against PL mediated 4-MUO hydrolysis. *Left*: the dose-dependent inhibition curve. *Middle*: the Lineweaver–Burk plot. *Right*: the second plot of slopes from the Lineweaver–Burk plots. All data represent the mean of triplicate determinations.

In order to explore the inhibitory mechanism of two dual-target potent inhibitors (**39** and **41**) on PL and hCES1A, inhibitory kinetic analysis was carried out. As shown in Figures S1 and S2, the dose-response curves of compounds **39** and **41** were not affected by the pre-incubation times, indicating that compounds **39** and **41** were reversible inhibitors against PL and hCES1A. Then, the inhibitory kinetics of compounds **39** and **41** against hCES1A were characterised in HLM. As shown in Figure 2(A,C), the results clearly show that both **39** and **41** inhibit the hCES1A-mediated hydrolysis of NLMe in HLM in a competitive manner, and the inhibition constants (*K*_i_) of **39** and **41** were calculated 0.043 µM and 0.019 µM, respectively. In addition, the inhibitory kinetics of compounds **39** and **41** against PL were characterised in PL. As shown in Figure 2(B,D), both **39** and **41** inhibited PL-mediated 4-MUO hydrolysis through a mixed manner, and the inhibition constants (*K*_i_) of **39** and **41** were calculated 1.45 µM and 0.58 µM, respectively.

### 3.7. Specificity of 39 and 41 towards hCES1A and PL over other human serine hydrolases

In view of the overlap of mammalian serine hydrolases, it is necessary to study the specificity of **39** and **41** on hCES1A and PL t over other human serine hydrolases. In this study, three other human serine hydrolases (hCES2A, DPP-IV and BuChE) were used to study the specificity of the dual-target inhibitors. As shown in Table 4, both compounds **39** and **41** were found with good selectivity over hCES2A and high selectivity over BuChE and DPP-IV. These indicate that compounds **39** and **41** are selective inhibitors of hCES1A and PL.

**Table 4.** The inhibitory effects of **39** and **41** towards five serine hydrolases.

Compound	Target enzyme	Substrate	IC_50_ (µM)	*K*_i_ (µM)	Inhibition mode
**39**	PL	4-MUO	2.13 ± 0.19	1.45	Mixed
	hCES1A	NLMe	0.055 ± 0.006	0.059	Competitive
	hCES2A	FD	6.02 ± 0.75	–	–
	BuChE	GP-BAN	>100	–	–
	DPP-IV	BTCH	>100	–	–
**41**	PL	4-MUO	0.75 ± 0.12	0.58	Mixed
	hCES1A	NLMe	0.014 ± 0.002	0.019	Competitive
	hCES2A	FD	5.02 ± 0.88	–	–
	BuChE	GP-BAN	>100	–	–
	DPP-IV	BTCH	>100	–	–

Data were shown as mean ± SD (*n* = 3).

### 3.8. Docking simulation

**Figure 3.**
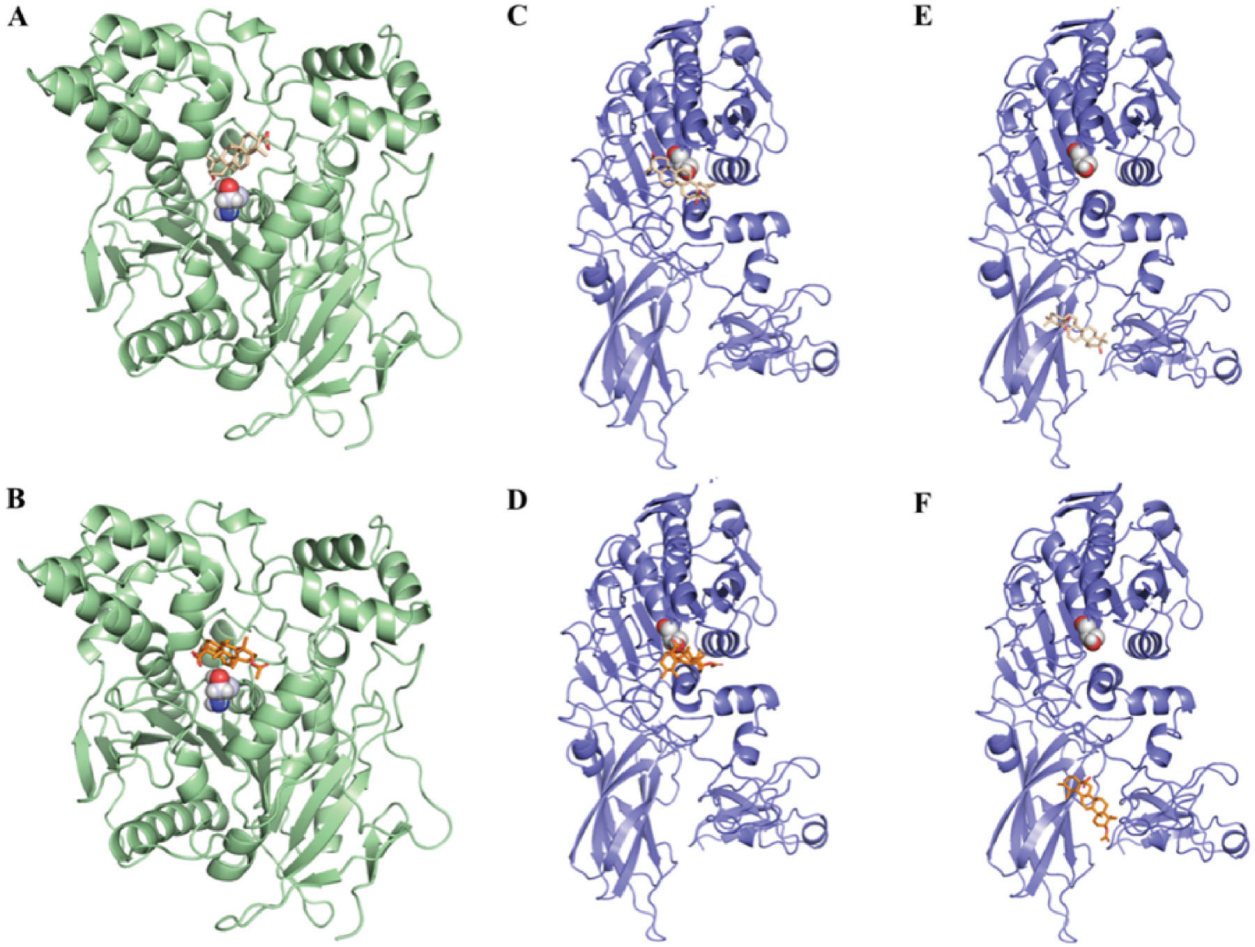
Overview of compounds **39** (A) and **41** (B) docked into the activity pocket of hCES1A. The stereo view of PL docked with compound **39** at site I (C) and site II（D); The stereo view of PL docked with compound **41** at site I (E) and site II (F).

In order to gain insight into the interaction mode between inhibitors (compounds **39** and **41**) and targets (PL and hCES1A), molecular docking simulations were carried out, respectively. The binding modes between compound **39**/**41** and hCES1A were explored firstly. As shown in Figure 3 and Table S1, both **39** and **41** could be well docked into the catalytic cavity of hCES1A. Compared to original ligand homotropine in crystal structure (PL, PDB: 1ETH; hCES1A, PDB: 1MX5), they occupy the position of homotropine, and could block the active site more completely, and the predicted binding energy were −9.4 kcal/mol and −9.6 kcal/mol, respectively. The detailed interaction (Figure S3) shows that their main mode of action was hydrophobic interaction. In addition, the binding poses between compound **39**/**41** and PL were explored as shown in Figure 3 and Table S1. At Site I (the Ser-153 for active site), hydrophobic interaction was the main interaction, and the energy were −7.4 kcal/mol and −7.5 kcal/mol, respectively. Both compounds **39/41** and ethylene glycol monooctyl ether (TGME), the original ligand of crystal structure, have hydrophilic and hydrophobic groups at both ends. The carboxyl in **39/41** tend to point to the solvent and occupy around the pocket to affect substrate entry. In addition, at Site II (the surface between lipase and colipase for interface site of PL), compound **38** can form hydrogen bond with Arg-65, and compound **41** can interact with Arg-65 and Lys-42 through hydrogen bonds (Figure S4), with energies of −8.6 kcal/mol and −8.6 kcal/mol, respectively. Colipase interacts with the lipase C-terminal domain (C domain) and with the flap (a surface loop from the N-terminal domain), so we speculated that binding at site II may affect flap movement and thus substrate binding.

### 3.9. Inhibit the formation of lipid droplets in 3T3-L1 cells

**Figure 4.**
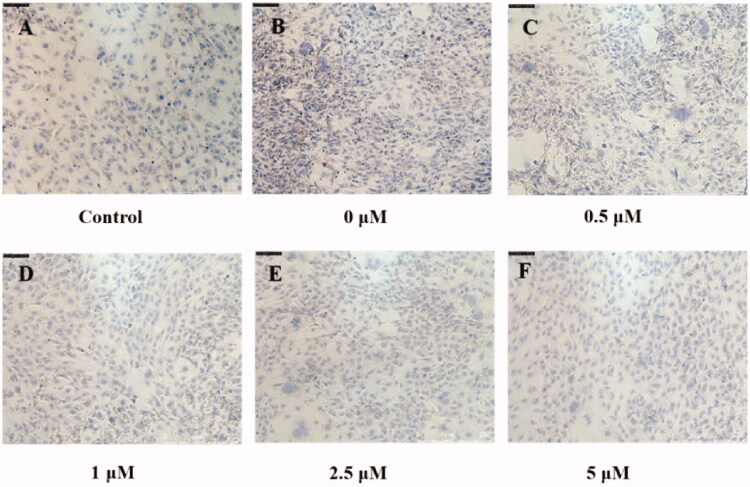
Oil red O staining results of 3T3-L1 cells (A) normally cultured and (B–F) differentiation culture treated with compound **39** (0–5 μM) (magnification, 10×).

**Figure 5.**
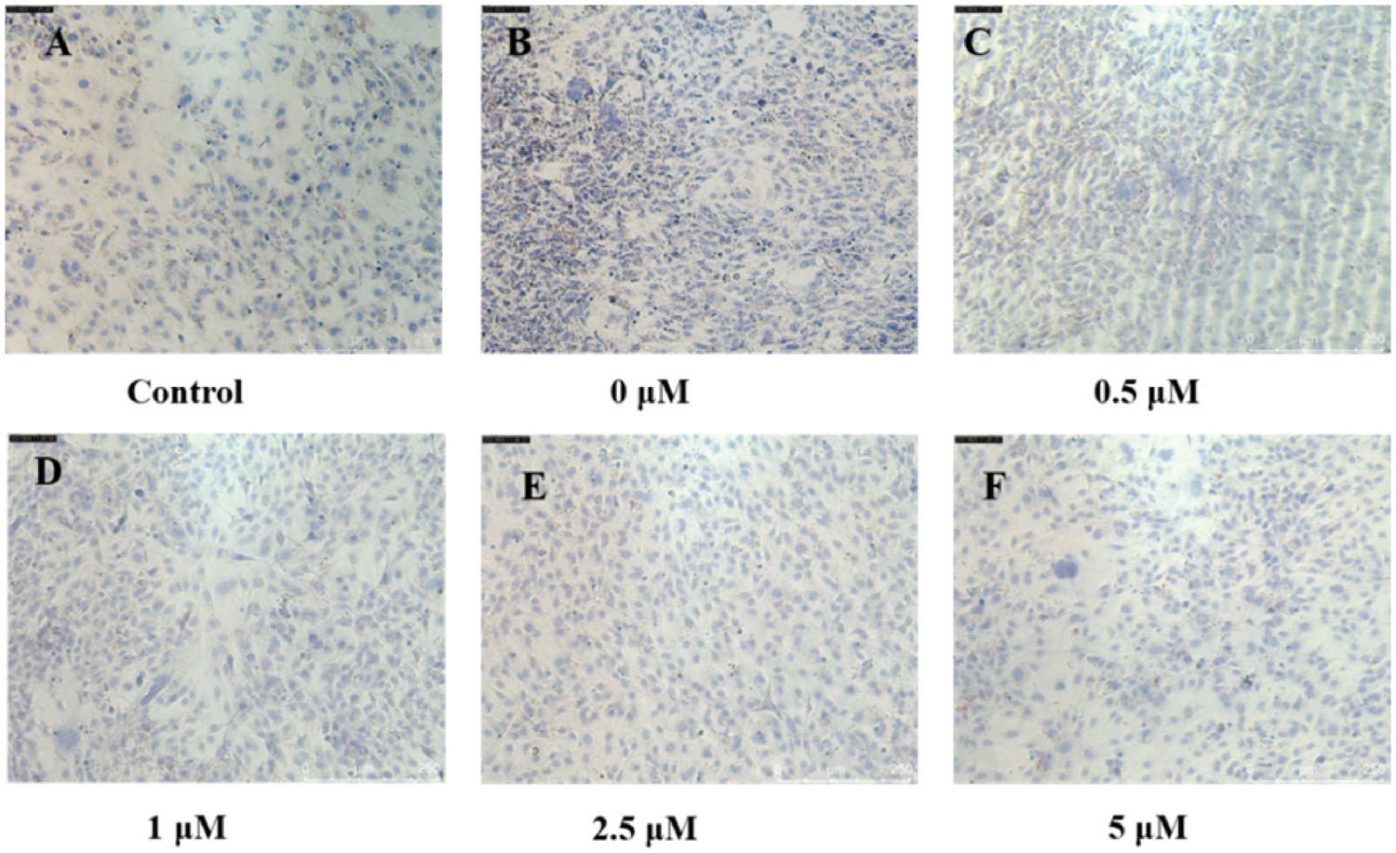
Oil red O staining results of 3T3-L1 cells (A) normally cultured and (B–F) differentiation culture treated with compound **41** (0–5 μM) (magnification, 10×).

In order to study the effects of dual-target inhibitors compounds **39** and **41** on the formation of lipid droplets during the induction of 3T3-L1 mouse preadipocytes into adipocytes, compounds **39** and **41** were added for pre-treatment before cell induction. First, the cell viability test results show that compounds **39** and **41** are almost non-toxic to 3T3-L1 below 20 µM and 10 µM, respectively (Figure S5). Next, compounds **39** and **41** at different concentrations were mixed with an inducer that induces adipocyte differentiation, and incubated with the cells for 96 h. After culturing with compounds **39** and **41** in normal medium for 96 h, the medium was changed every 2 days, and then the cells were stained with Oil Red O dye. The results showed that the lipid droplets in the induced 3T3-L1 cells (Figure 4(B)) were significantly higher than those in the uninduced control group (Figure 4(A)). In addition, almost no lipid droplets were formed in the cells treated with the high concentration (5 µM) of compounds **39** and **41** (Figures 4(F) and 5(F)). The formation of lipid droplets in cells treated with low concentrations (<1 µM) were similar to that of uninduced controls. In addition, it can be seen from Figures 4 and 5(C–F) that the lipid droplet content of compounds **39**/**41** gradually decreases from low concentration to high concentration. The results indicate that two dual-target inhibitors could inhibit the formation of lipid droplets in adipocytes induced by preadipocytes.

## 4. Conclusion

In summary, a series of natural triterpenoids were collected and their inhibitory effects against PL and hCES1A were assayed using 4-MUO and NLMe as substrate probe, respectively. Two natural pentacyclic triterpenoid OA and UA were found to display both good inhibitory effects on PL and hCES1A, and good selectivity over hCES2A. Thus, 14 compounds based on the UA and UA skeletons were synthesised and evaluated. Structure-activity relationship (SAR) analysis of these compounds revealed that 2-enol and 3-ketal moiety are beneficial for PL and hCES1A inhibition, and smaller 3-acetyl group is beneficial for PL inhibition, while bigger 3-O-β-carboxypropionyl group is beneficial for hCES1A. In addition, compounds **39** (OA derivative with 2-enol and 3-ketal moiety) and **41** (OA derivative with acetyl group at the C-3 site) displayed potent inhibitory effects against both PL and hCES1A. Furthermore, compounds **39** and **41** exhibited good selectivity over other human serine hydrolases including hCES2A, BChE and DPP-IV. Inhibitory kinetics and molecular docking studies demonstrated that both compounds **39** and **41** were effective mixed inhibitors of PL, while competitive inhibitors of hCES1A. Further investigations demonstrated that both compounds **39** and **41** could inhibit adipocyte adipogenesis induced by mouse preadipocytes. Collectively, our findings suggest that triterpenoids are good choices for design and development of PL and hCES1A inhibitors, while compounds **39** and **41** hold great promise for development of novel PL and hCES1A dual-target inhibitors to treat with related metabolic diseases.

## Supplementary Material

Supplemental Material
