# Supplementary material for: Discovery of triterpenoids as potent dual inhibitors of pancreatic lipase and human carboxylesterase 1
Source: J Enzyme Inhib Med Chem. 2022 Jan 31;37(1):629–40. doi: 10.1080/14756366.2022.2029855 (PMC8812735; doi:10.1080/14756366.2022.2029855)
Supplement: Supplemental Material [file IENZ_A_2029855_SM1680.pdf]

Supplementary Materials

for

Discovery of triterpenoids as potent dual inhibitors of pancreatic lipase and human carboxylesterase 1

Jing Zhang [a,#], Qiu-Sha Pan [a,#], Xing-Kai Qian [a, c], Xiang-Lu Zhou [a], Ya-Jie Wang [a], Rong-Jing He [a], Le-Tian Wang [a], Yan-Ran Li [a], Hong Huo [d], Cheng-Gong Sun [b], Lei Sun [b, **], Li-Wei Zou [a, *] and Ling Yang [a]

[a] Institute of Interdisciplinary Integrative Medicine Research, Shanghai University of Traditional Chinese Medicine, Shanghai, 201203, China

[b] The Second Hospital of Dalian Medical University, Dalian, 116023, China

[c] Translational Medicine Research Center, Guizhou Medical University, University Town, Guian New District, 550025, Guizhou, China.

[d] Dalian Institute of Chemical Physics, Chinese Academy of Sciences, Dalian, 116023, China

*Corresponding author.

** Corresponding author.

E-mail addresses: chemzlw@163.com (L.-W. Zou), 417186487@qq.com (L. Sun)

# These authors contributed equally to this work.

Contents:

Fig. S1 Non-covalent interaction analysis of compound **39** and **41** with hCES1A-------------3

Fig. S2 Non-covalent interaction analysis of compound **39** and **41** with PL------------------3

Fig. S3 2D interactions compound **39** and **41** with hCES1A------------------------------------3

Fig. S4 2D interactions compound **39** and **41** with PL --4

Fig. S5 Cell viability of compound **39** and **41** ---4

Table S1 The detailed interactions between compounds **39/41** and hCES1A/PL---------------5

Synthesis of triterpenoids derivatives--6

NMR spectrums of the triterpenoids derivatives ---7

Reference---21

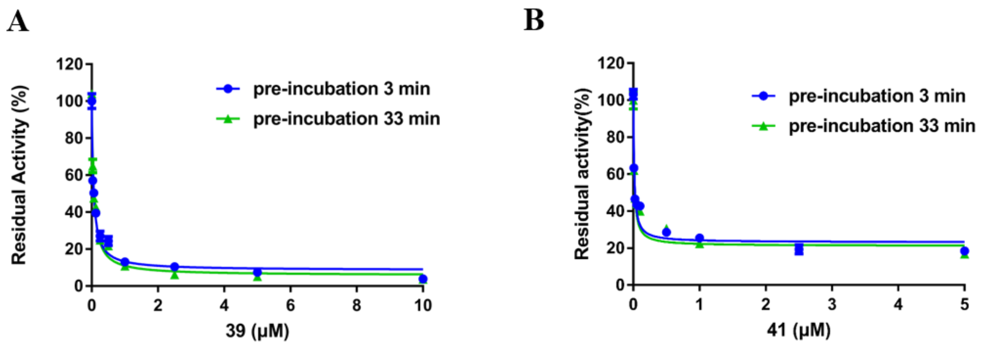

Fig. S1 Non-covalent interaction analysis of compound **39** and **41** with hCES1A. Data were shown as mean ± SD (n=3).

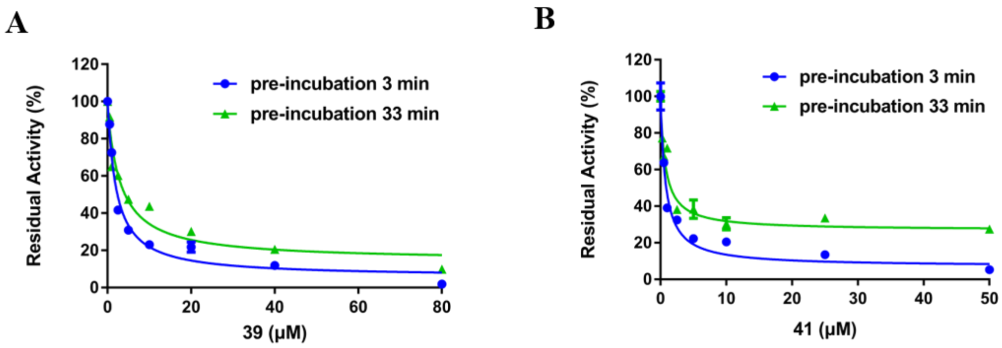

Fig. S2 Non-covalent interaction analysis of compound **39** and **41** with PL. Data were shown as mean ± SD (n=3).

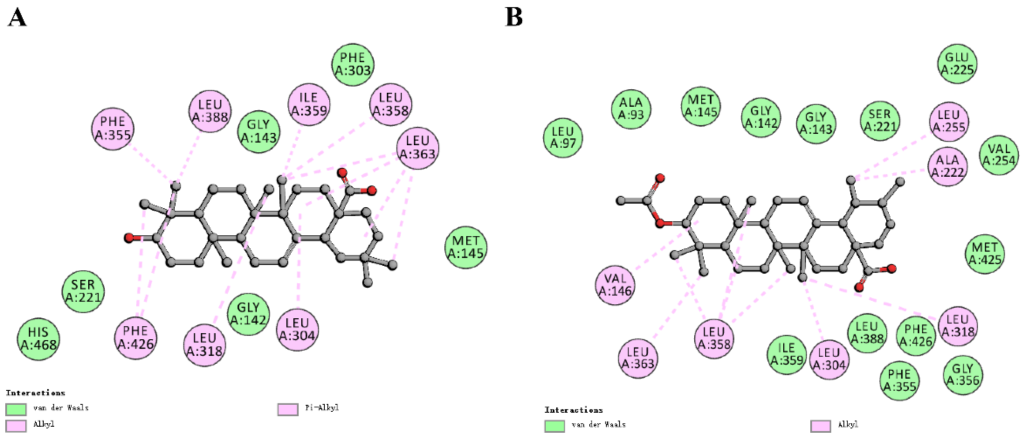

Fig. S3 2D interactions between compound **39** (A), compund **41** (B) and the amino acid residues surrounding to the catalytic site of hCES1A.

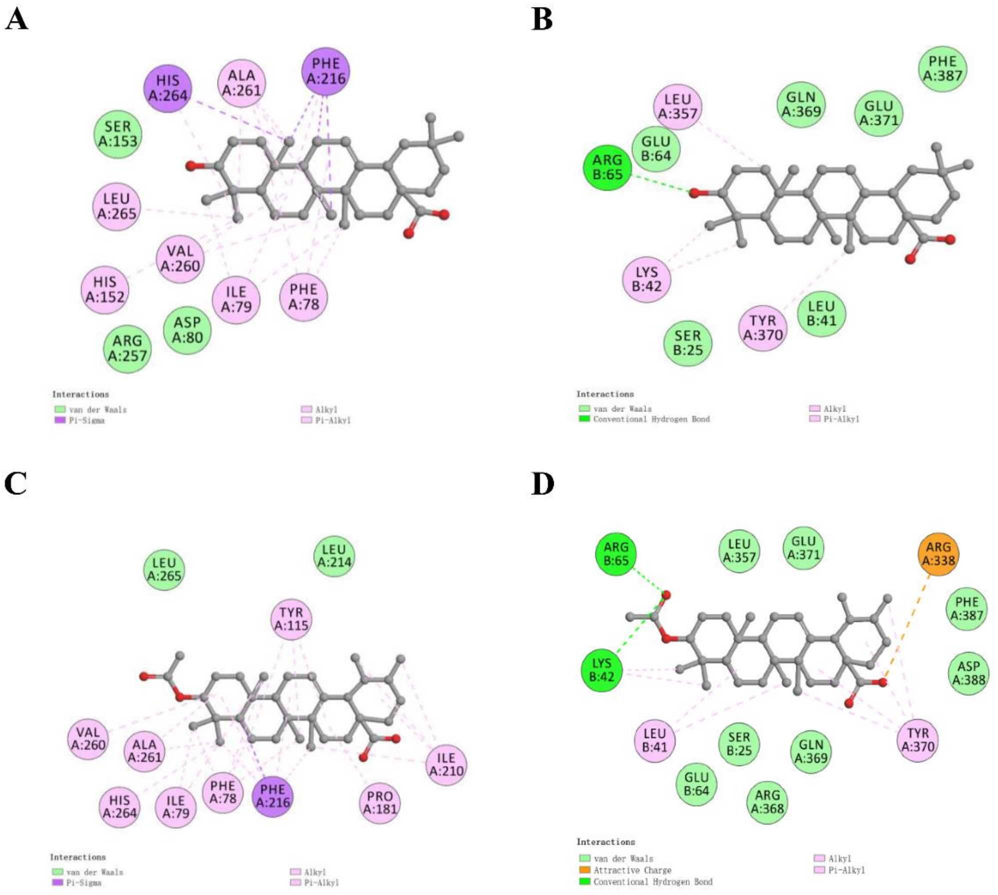

Fig. S4 2D interactions between compound **39** and PL at site 1 (A) and site 2 (B); 2D interactions between compound **41** and PL at site 1 (C) and site 2 (D).

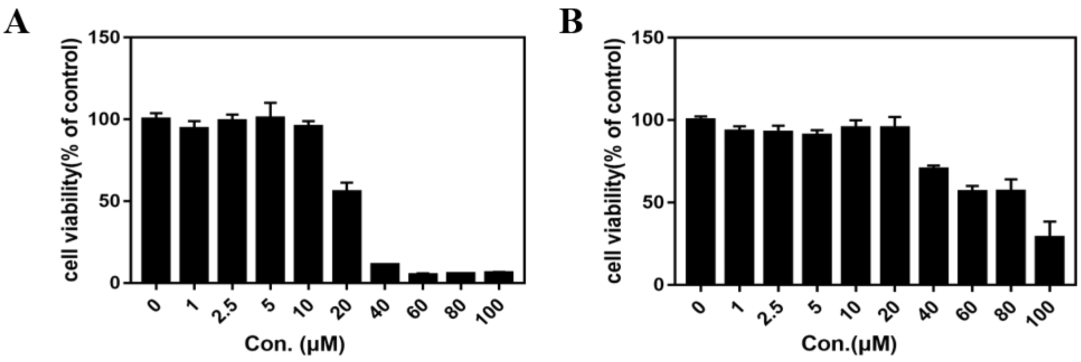

Fig. S5 Cell viability of compound **39** (A) and compound **41** (B) at different concentrations (0-100μM) in 3T3-L1.

Table S1. The detailed interactions between compounds **38/41** and hCES1A/PL

Enzyme	Compounds	Site	Hydrophobic interaction	Hydrogenbond	Attractivecharge
hCES1A	39	\	Phe 303, Phe 355, Phe 426, Leu 304, Leu 318, Leu 353, Leu 363,Leu3 88, Arg 257, Ser 221, His 468, Gly 142, Gly 143, Met 145, Ile 359	\	\
	41	\	Phe 355, Phe 426, Leu 255, Leu 304, Leu 318, Leu 353, Leu 363, Leu 388, Arg 257, His 468, Gly 142, Gly 143, Gly 356, Met 145, Met 425, Ser 221, Val 126, Val 254, Ile 359	\	\
PL	39	Site I	Phe 78, Phe 216, His 152, His 264, Asp 80, Arg 257, Val 260, Ala 261, Leu 265, Ile 79, Ser 153	\	\
	39	Site II	Leu 357, Leu 41, Glu 64, Gln 369, Glu 371, Lys 42, Ser 25, Tyr 370, Phe 387	Arg 65*	\
	41	Site I	Leu 265, Leu 214, Tyr 115, Ile 79, Ile 210, Phe 78, Phe 216, Val 260, Ala 261, His 264, Pro 181	\	\
	41	Site II	Leu 357, Leu 41, Phe 387, Tyr 370, Glu 64, Gln 369, Glu 371, Ser 25, Arg 368, Asp 388	Arg 65*, Lys 42*	Arg 338

*represent chain B of PL

Synthesis of triterpenoids derivatives

Compound **30, 32, 33, 36-38, 40, 42** were prepared according to the literature[1, 2]. Compound **31, 34, 35, 39, 41** and **43** were prepared according to the following procedure.

Synthesis of compound **31**

To a stirred solution of OA (457 mg, 1.0 mmol) in pyridine (7.5 mL), acetic anhydride (5.0 mL) was added dropwise at 0°C. The resulting solution was stirred at room temperature for 24 h and then poured into ice water (50 mL), resulting in the compound 41 (432 mg, 86%) as the white solid precipitate.

Synthesis of compound **34**

To a stirred solution of oleanoic acid (228.4 mg, 0.5 mmol) in dichloromethane (10 mL), *n*-butyric anhydride (395.5 mg, 2.5 mmol) and DMAP (122.17 mg, 1.0 mmol) were added at 0°C. The resulting solution was stirred at room temperature for 24 h and then poured into ice water (50 mL). Organic phase was separated and aqueous layer was further extracted with dichloromethane (25 mL×2). The combined organic phase was washed with water (20 mL), brine (20 mL) and dried over sodium sulfate. After evaporation of the solvent, the crude residue was purified by column chromatography on silica gel (petroleum ether/ethyl acetate = 5/1) to give the compound **34** (169.1 mg, 64%) as a white solid.

Synthesis of compound **35**

The preparation was performed as described above for compound **34** starting from ursolic acid **5** (228.4 mg, 0.5 mmol) to give compound **35** (197.2 mg, 71%) as a white solid.

Synthesis of compound **39**

To a solution of oleanoic acid (456.7 mg, 1 mmol) in acetone (10 mL) was added Jones reagent (prepared from 107.9 mg of CrO_3) at 0 ºC over a period of 30 min till the brown color persisted. The resulting solution was stirred for further 30 min. Progress of the reaction was monitored by TLC. After completion of the reaction, isopropanol (0.5 mL) was added. After evaporation of the solvent, the crude residue was diluted with t-BuOH/THF (10/1, 10 mL) and then added t-BuOK (670 mg, 6.0 mmol) at room temperature. The resulting solution was stirred and refluxed for 10 h and then poured into 1M HCl aq. (10 mL). The aqueous phase was extracted with ethyl acetate (25

mL×3). The combined organic phase was washed with water (20 mL), brine (20 mL) and dried over sodium sulfate. After evaporation of the solvent, the crude residue was purified by column chromatography on silica gel (petroleum ether/ethyl acetate = 2/1) to give the compound **39** (159.3 mg, 34%) as a light yellow solid.

Synthesis of compound **43**

The preparation was performed as described above for compound **39** starting from ursolic acid (456.7 mg, 1 mmol) to give compound **43** (145.3 mg, 31%) as a light yellow solid.

Synthesis of compound **41**

The preparation was performed as described above for compound **31** starting from ursolic acid (457 mg, 1.0 mmol) to give compound **41** (456 mg, 92%) as a white solid.

NMR spectra for triterpenoids derivatives

Compound **30**

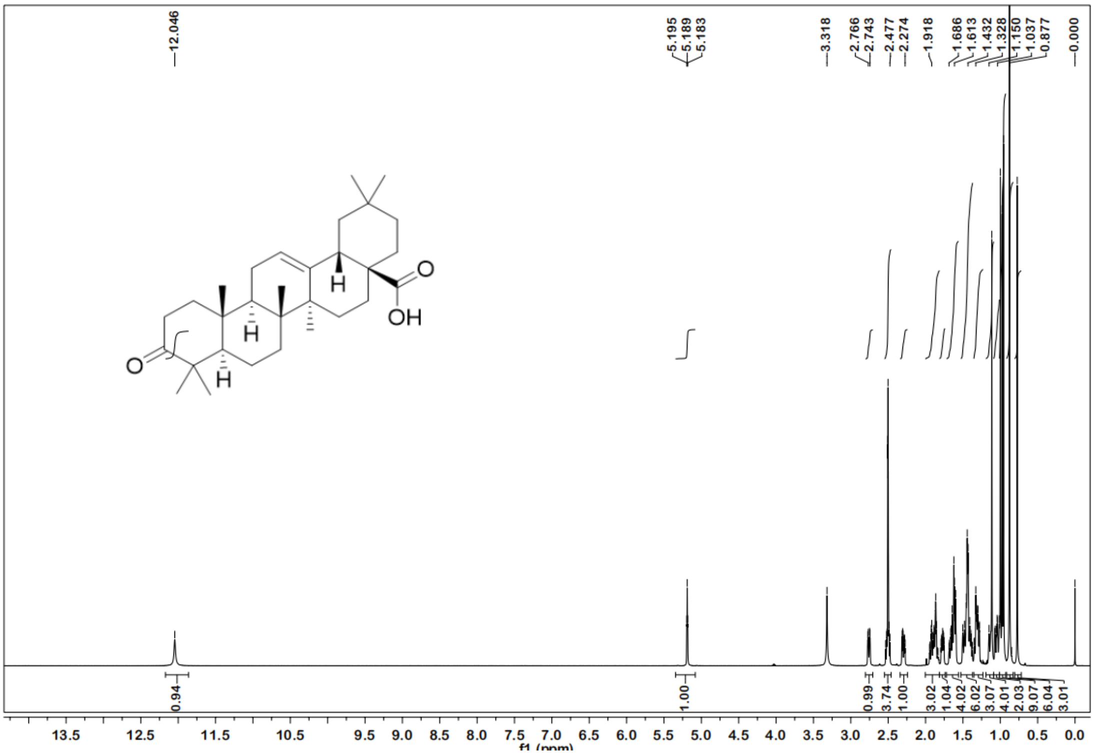

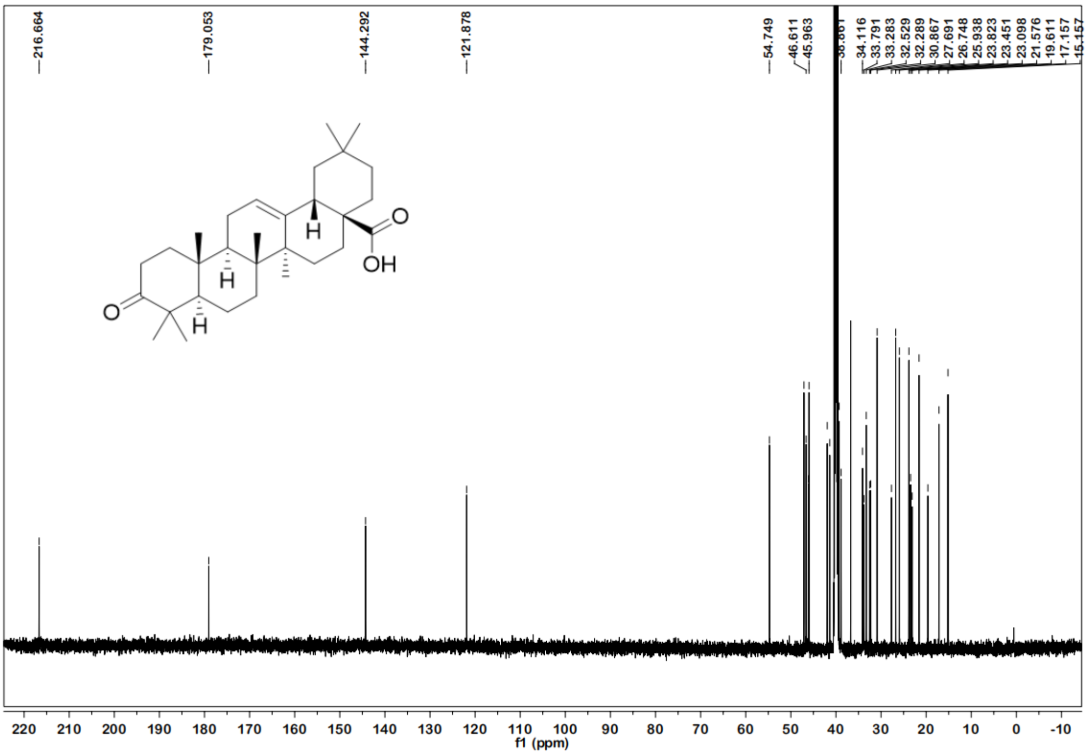

Compound **31**

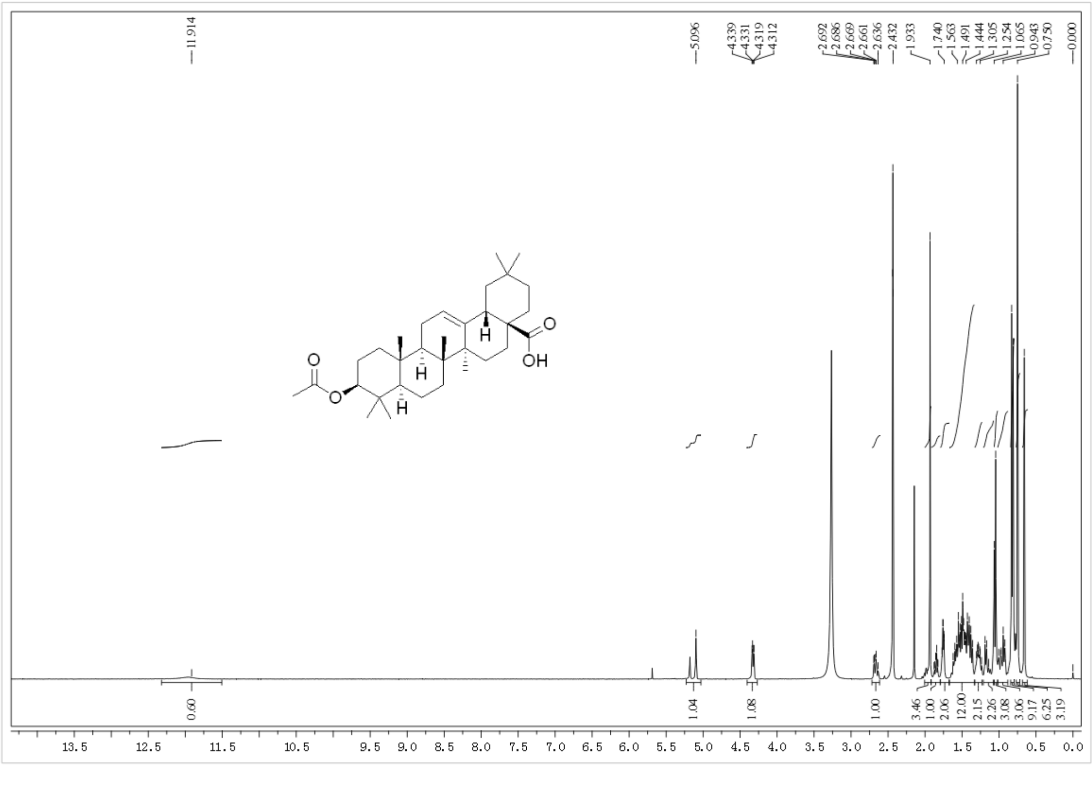

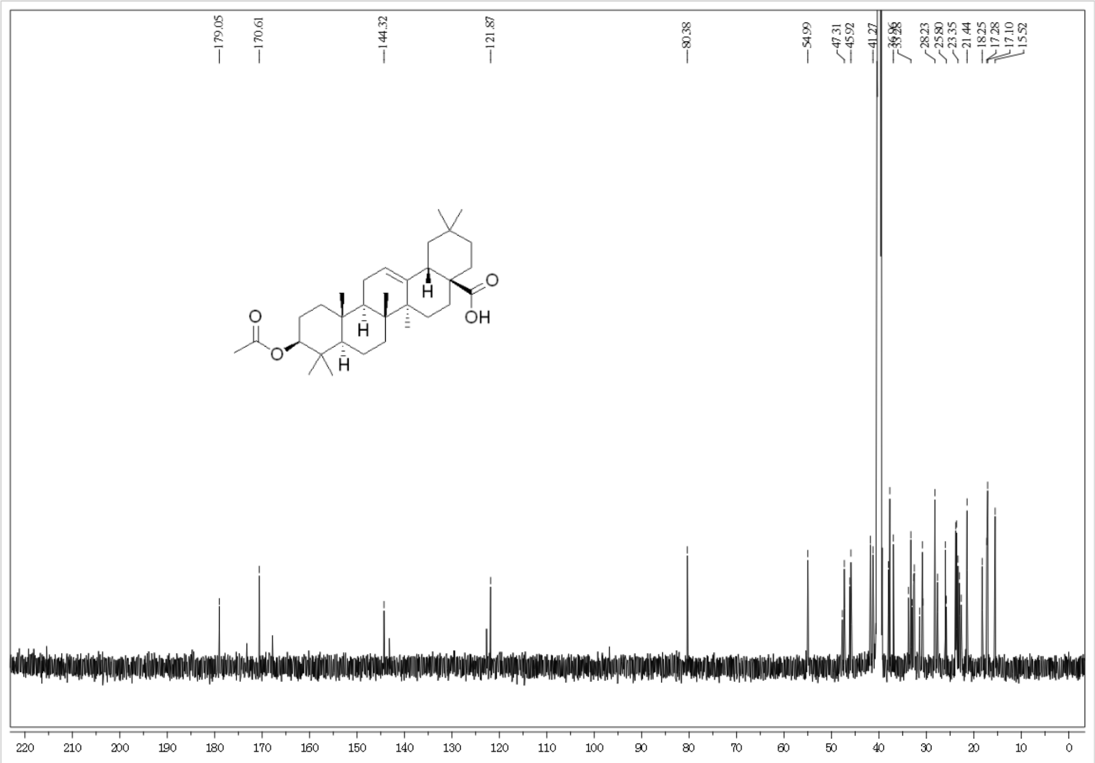
179.05
170.61
144.32
121.87
80.38
54.99
47.31
45.92
41.27
28.23
25.80
23.35
21.44
18.25
17.28
17.10
15.52
OH
O
H
220 210 200 190 180 170 160 150 140 130 120 110 100 90 80 70 60 50 40 30 20 10 0

Compound **32**

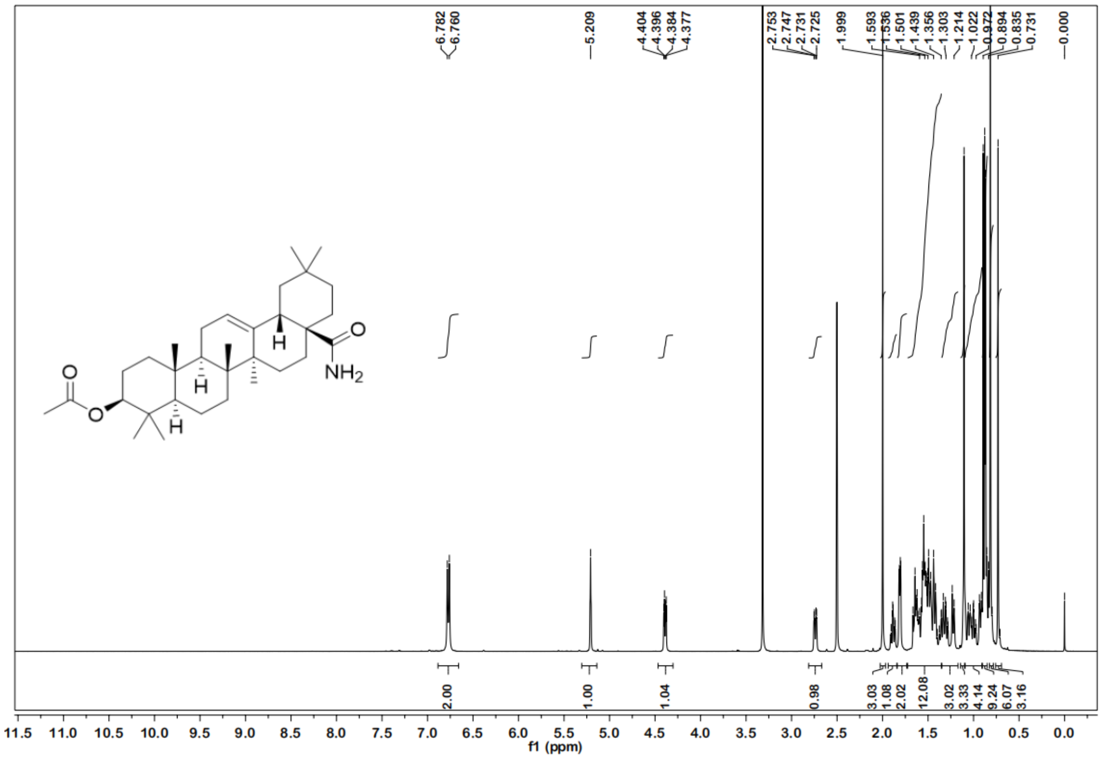
6.782
6.760
5.209
4.404
4.396
4.384
4.377
2.753
2.747
2.731
2.725
1.999
1.593
1.536
1.501
1.439
1.356
1.303
1.214
1.022
0.972
0.894
0.835
0.731
0.000
NH2
O
H
2.00
1.00
1.04
0.98
3.03
1.08
2.02
12.08
3.02
3.33
4.14
9.24
6.07
3.16
11.5 11.0 10.5 10.0 9.5 9.0 8.5 8.0 7.5 7.0 6.5 6.0 5.5 5.0 4.5 4.0 3.5 3.0 2.5 2.0 1.5 1.0 0.5 0.0
f1 (ppm)

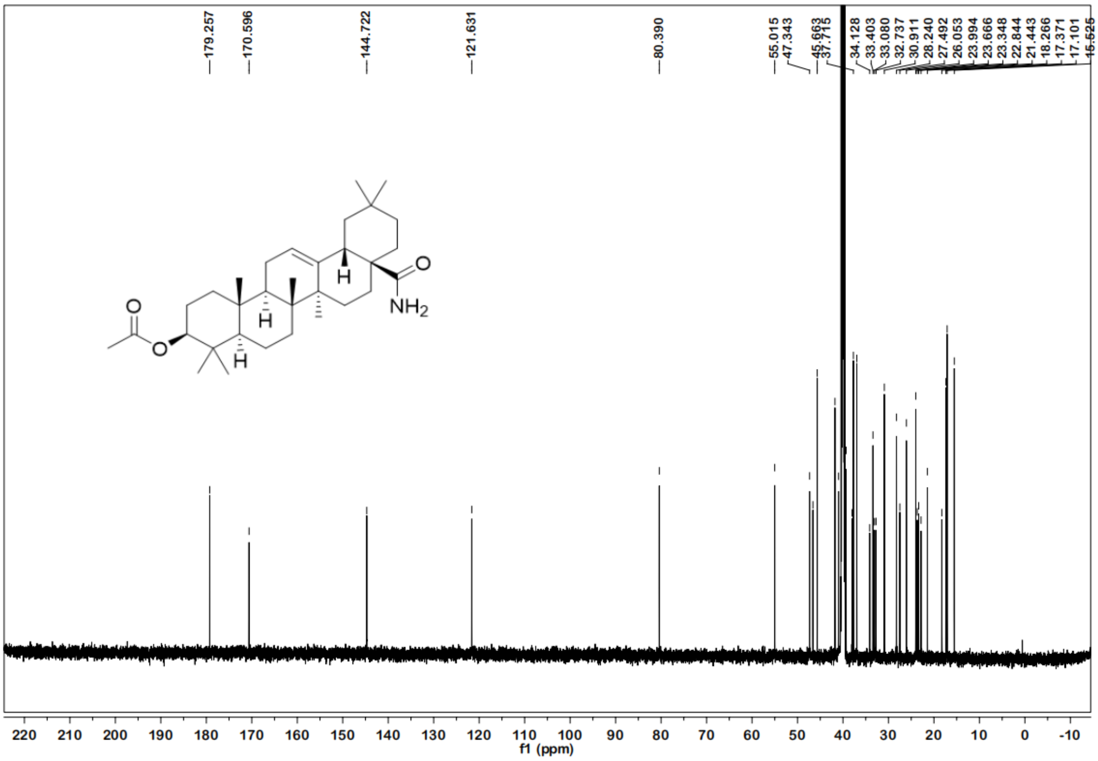
179.257
170.596
144.722
121.631
80.390
55.015
47.343
45.663
37.715
34.128
33.403
33.080
32.737
30.911
28.240
27.492
26.053
23.994
23.666
23.348
22.844
21.443
18.266
17.371
17.101
15.525
NH2
f1 (ppm)

Compound **33**

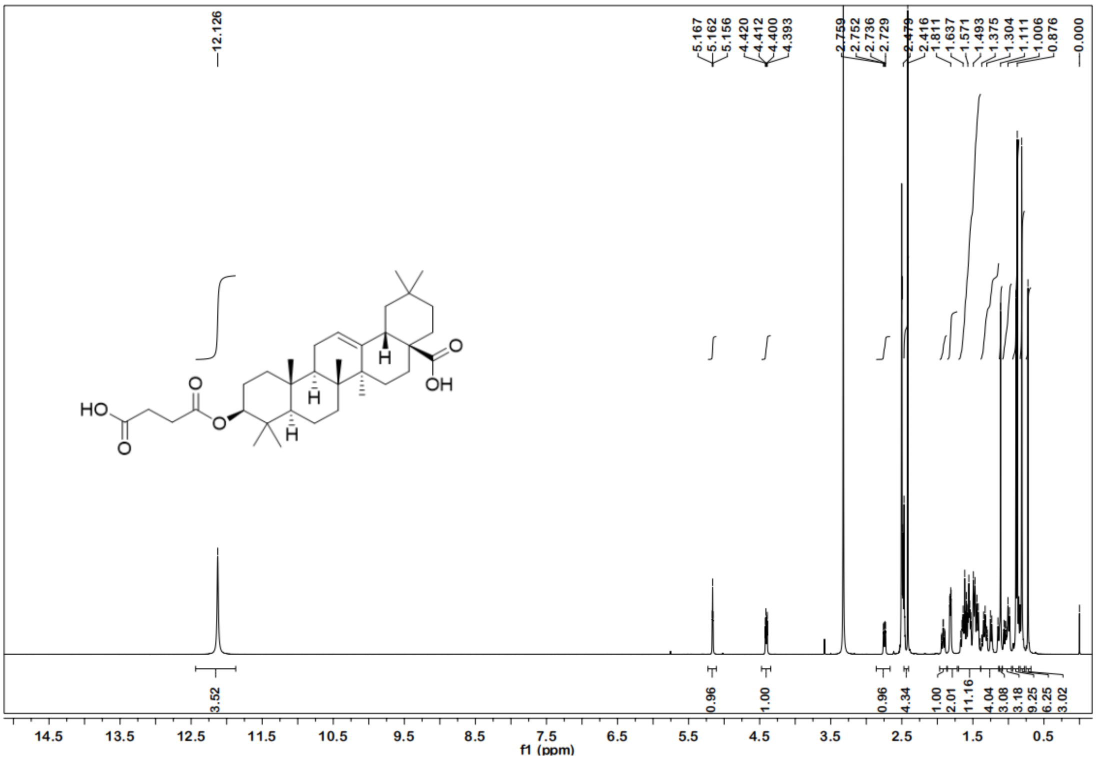
12.126
5.167
5.162
5.156
4.420
4.412
4.400
4.393
2.759
2.752
2.736
2.729
2.479
2.416
1.811
1.637
1.571
1.493
1.375
1.304
1.111
1.006
0.876
0.000
3.52
0.96
1.00
0.96
4.34
1.00
2.01
11.16
4.04
3.08
3.18
9.25
6.25
3.02
HO
OH
f1 (ppm)

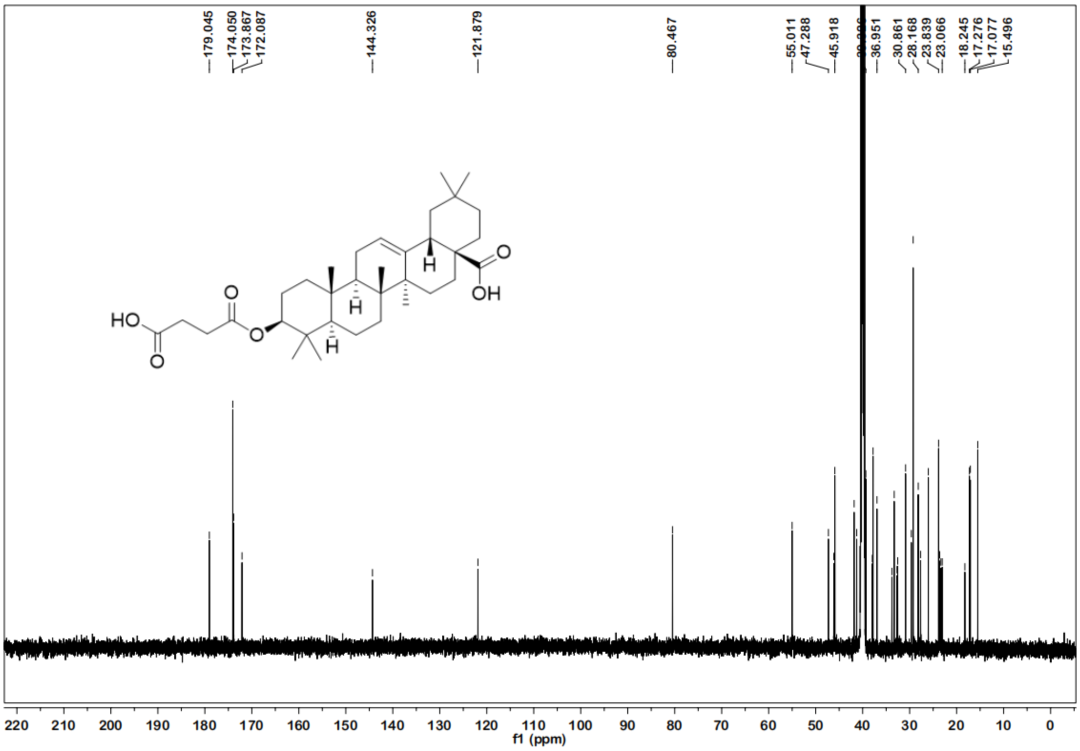
179.045
174.050
173.867
172.087
144.326
121.879
80.467
55.011
47.288
45.918
36.951
30.861
28.168
23.839
23.066
18.245
17.276
17.077
15.496
HO
O
OH
H
f1 (ppm)

Compound **34**

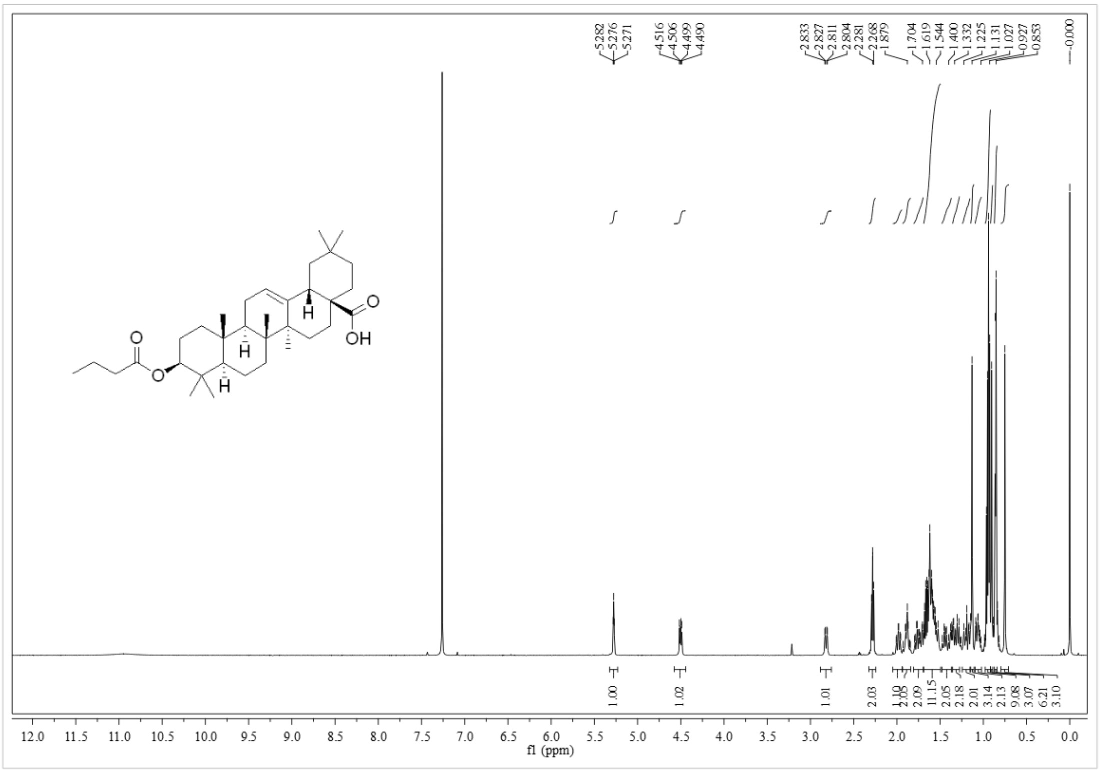
5.282
5.276
5.271
4.516
4.506
4.499
4.490
2.833
2.827
2.811
2.804
2.281
2.268
1.879
1.704
1.619
1.544
1.400
1.332
1.225
1.131
1.027
0.927
0.853
0.000
1.00
1.02
1.01
2.03
1.10
2.05
2.09
11.15
2.05
2.18
2.01
3.14
2.13
9.08
3.07
6.21
3.10
O
OH
H
f1 (ppm)

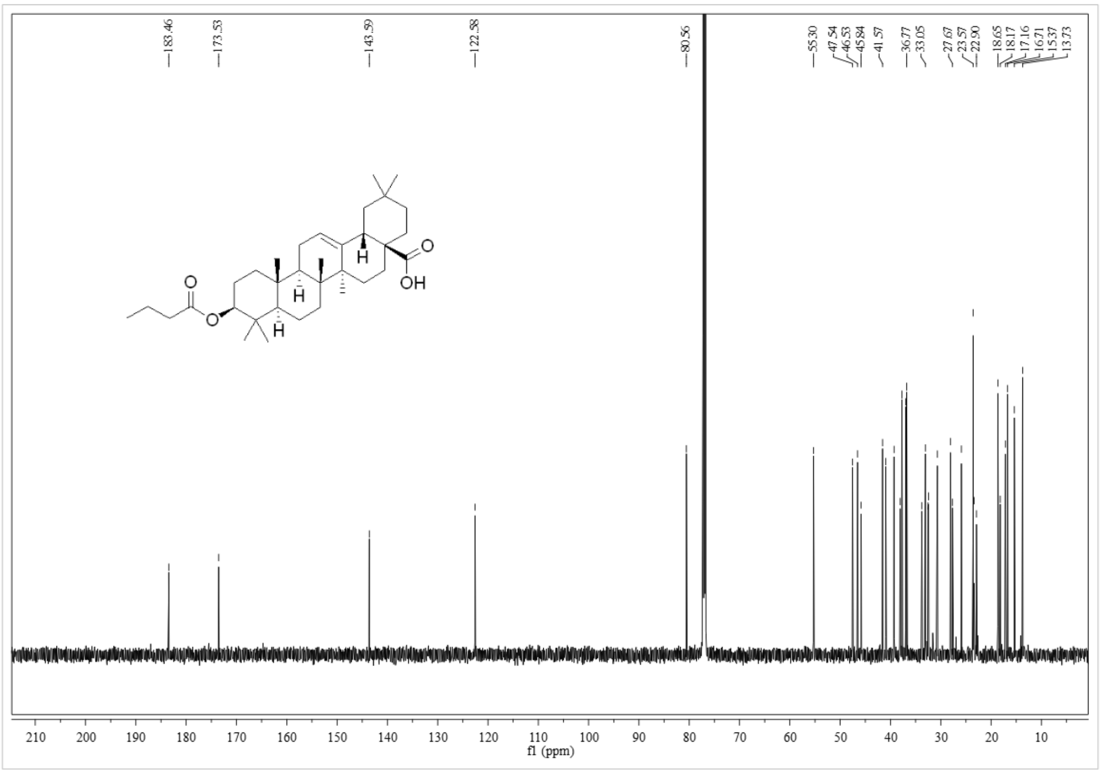

Compound **35**

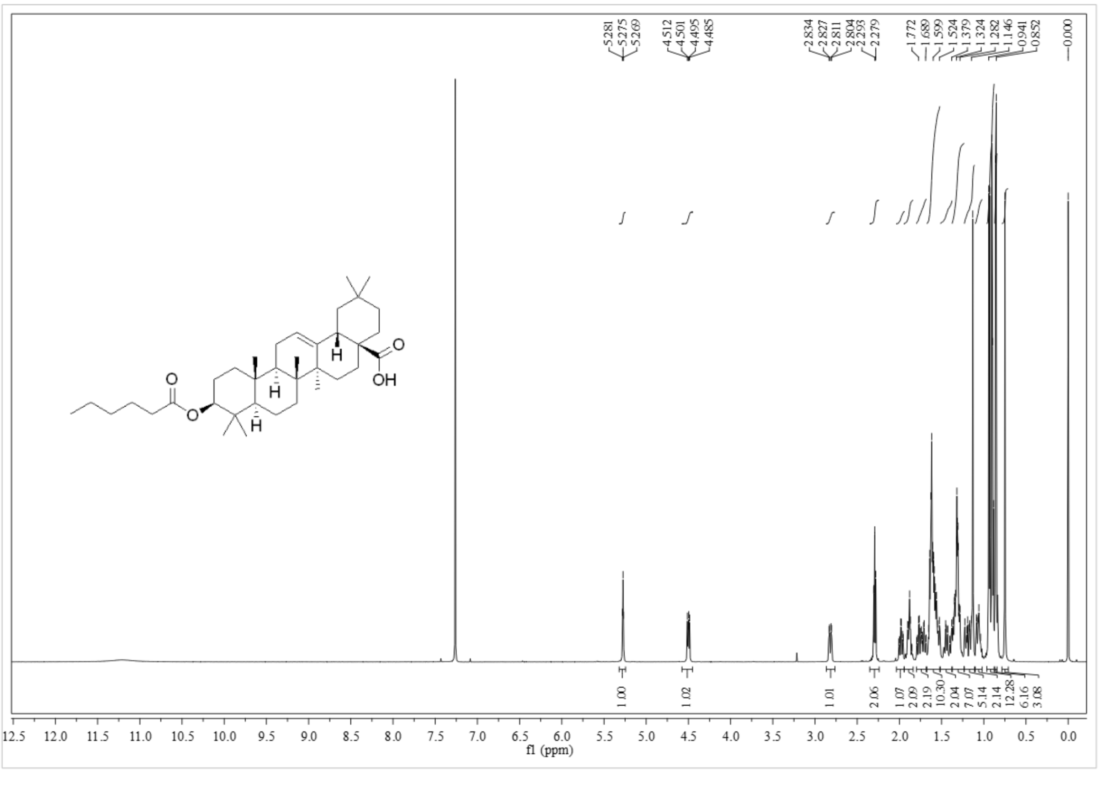

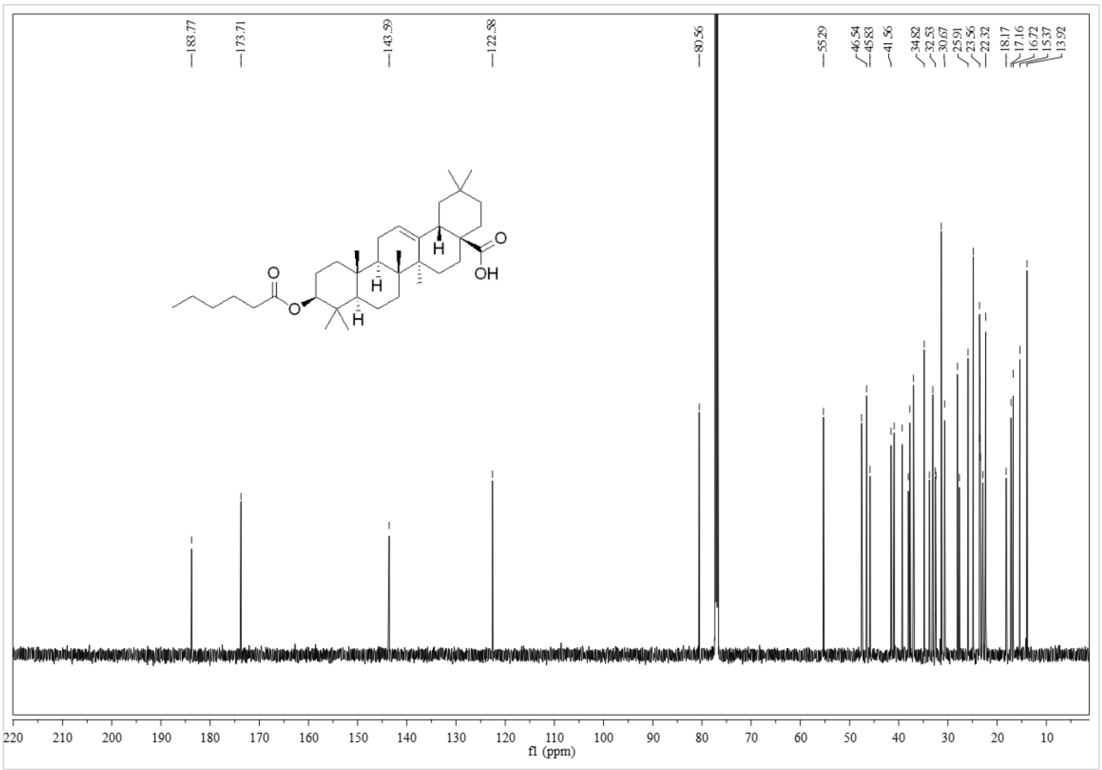

Compound **36**

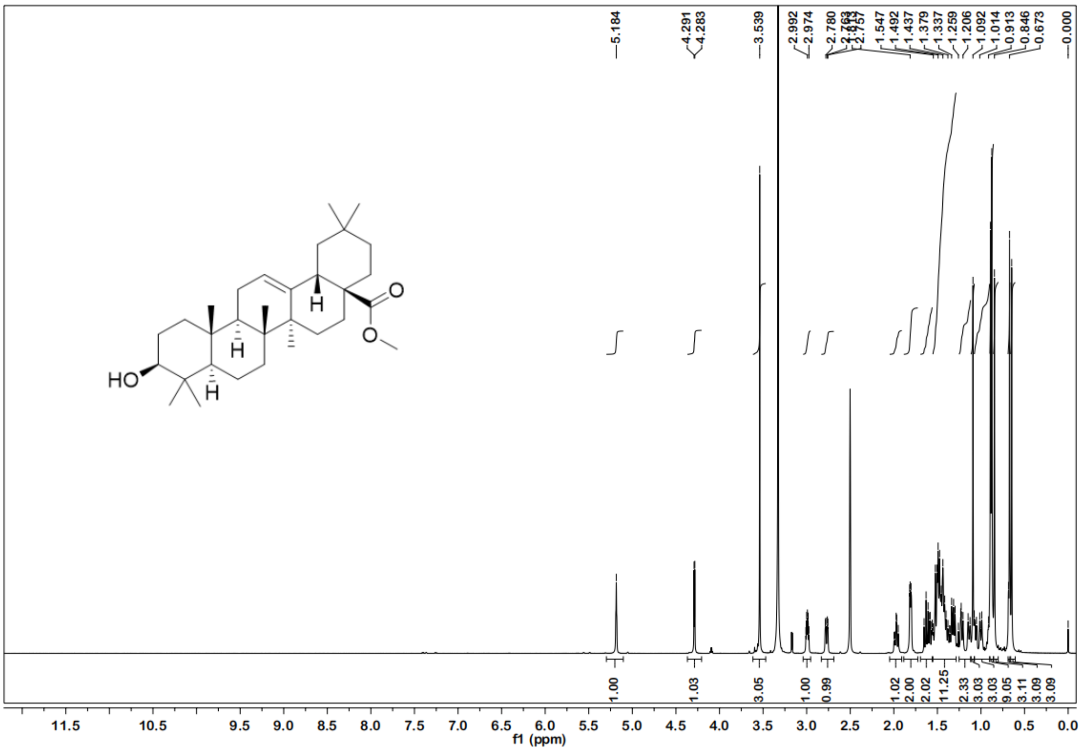

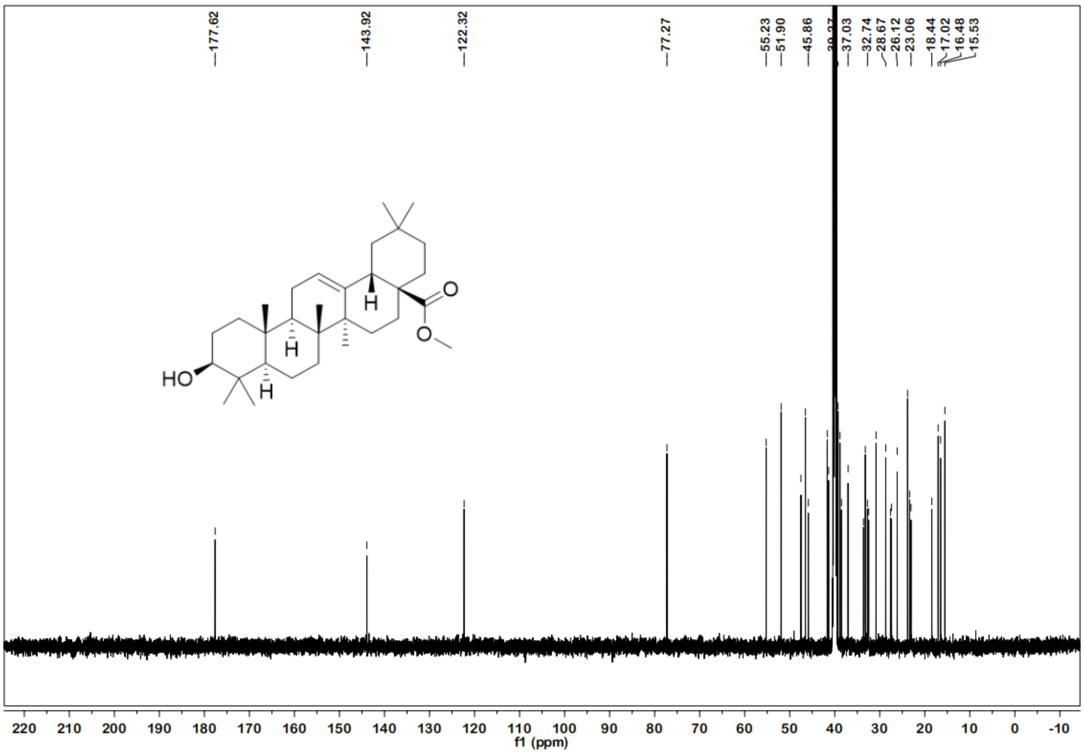
177.62
143.92
122.32
77.27
55.23
51.90
45.86
37.03
32.74
28.67
26.12
23.06
18.44
17.02
16.48
15.53
HO
H
O
220 210 200 190 180 170 160 150 140 130 120 110 100 90 80 70 60 50 40 30 20 10 0 -10
f1 (ppm)

Compound **37**

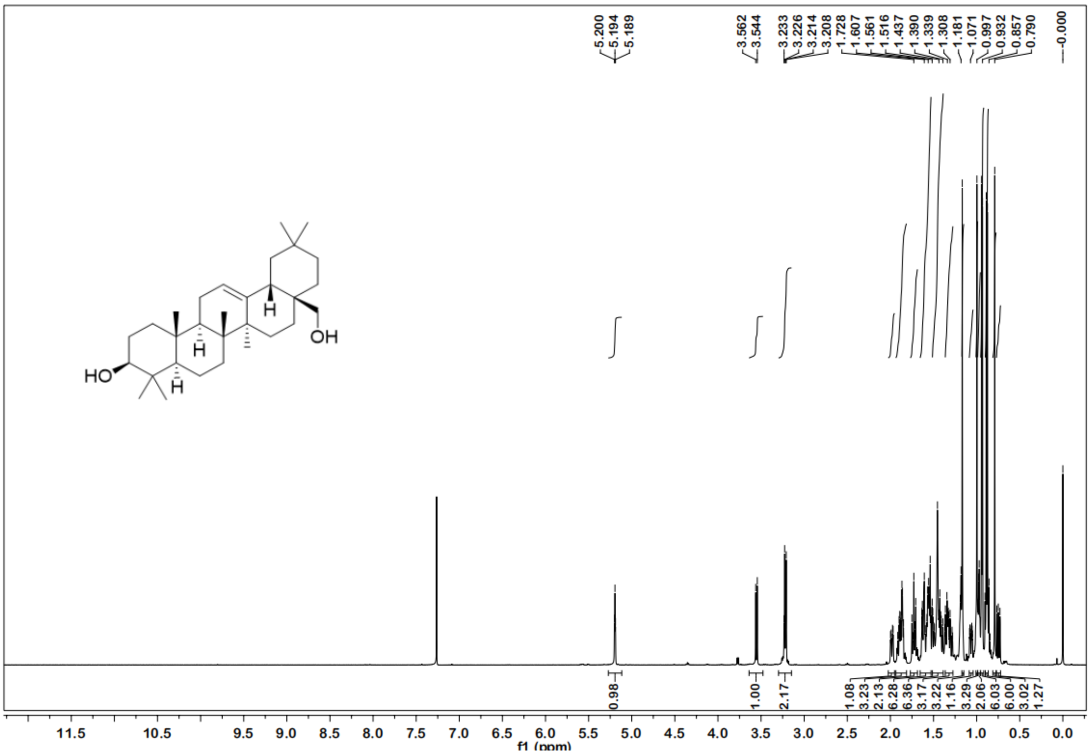
5.200
5.194
5.189
3.562
3.544
3.233
3.226
3.214
3.208
1.728
1.607
1.561
1.516
1.437
1.390
1.339
1.308
1.181
1.071
0.997
0.932
0.857
0.790
-0.000
HO
H
OH
0.98
1.00
2.17
1.08
3.23
2.13
6.28
6.36
3.17
3.22
1.16
3.29
2.06
6.03
6.00
3.02
1.27
11.5 10.5 9.5 9.0 8.5 8.0 7.5 7.0 6.5 6.0 5.5 5.0 4.5 4.0 3.5 3.0 2.5 2.0 1.5 1.0 0.5 0.0
f1 (ppm)

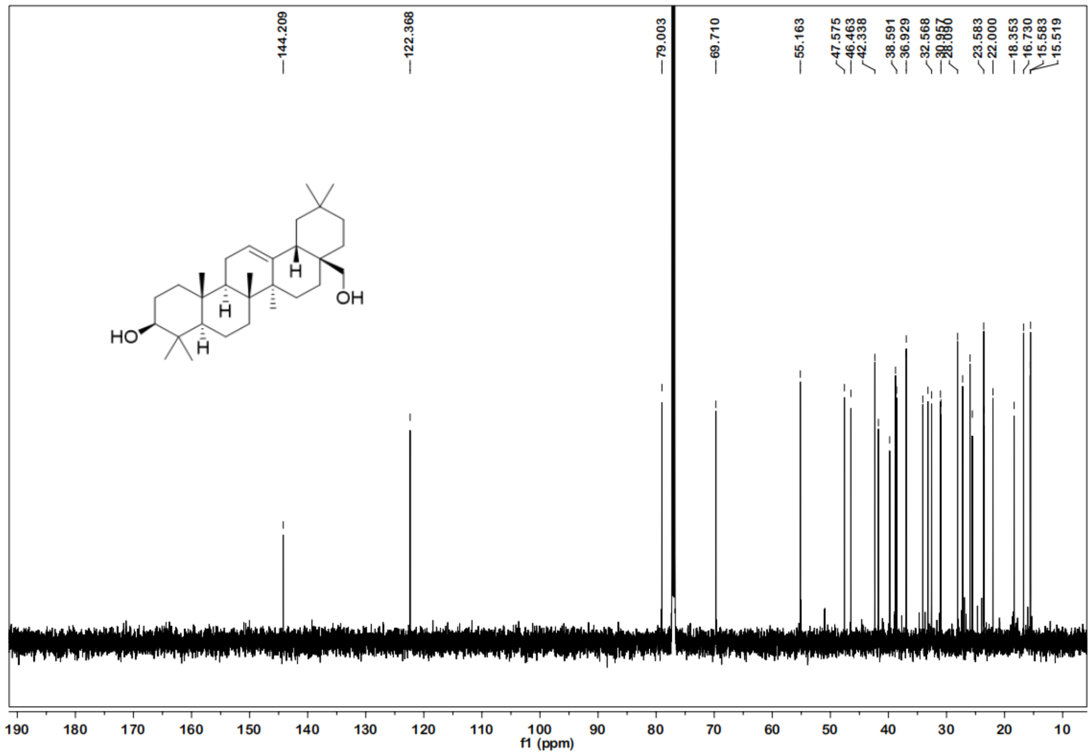

Compound **38**

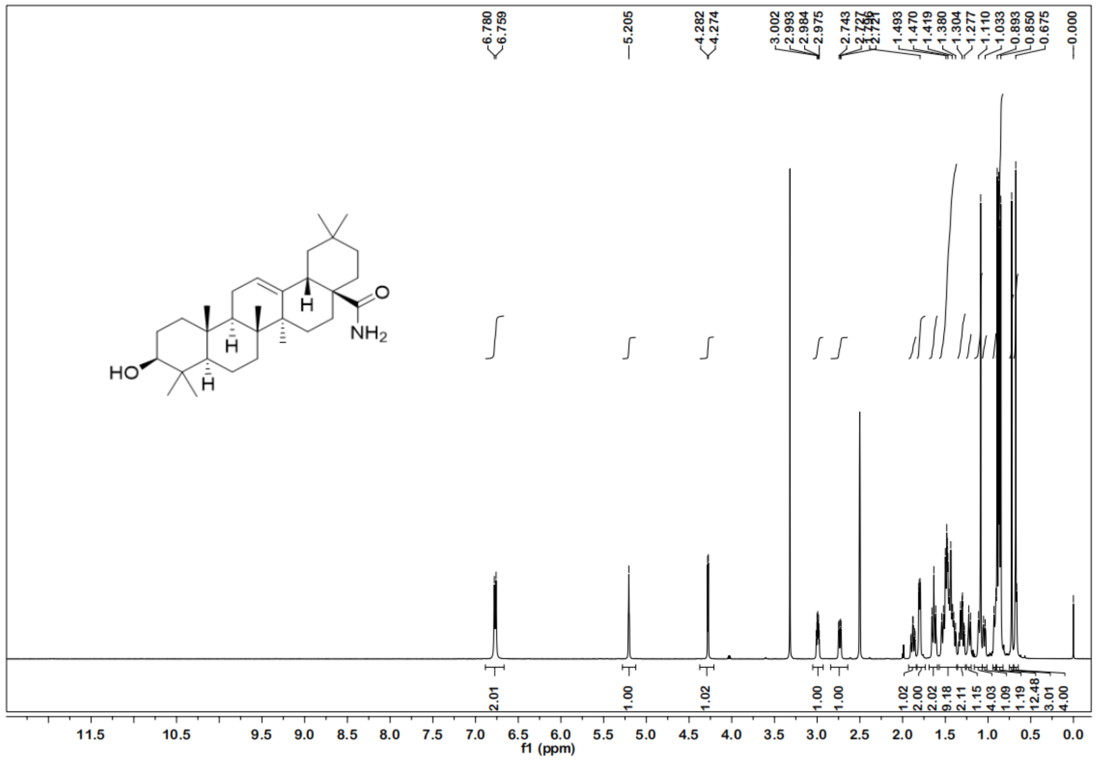

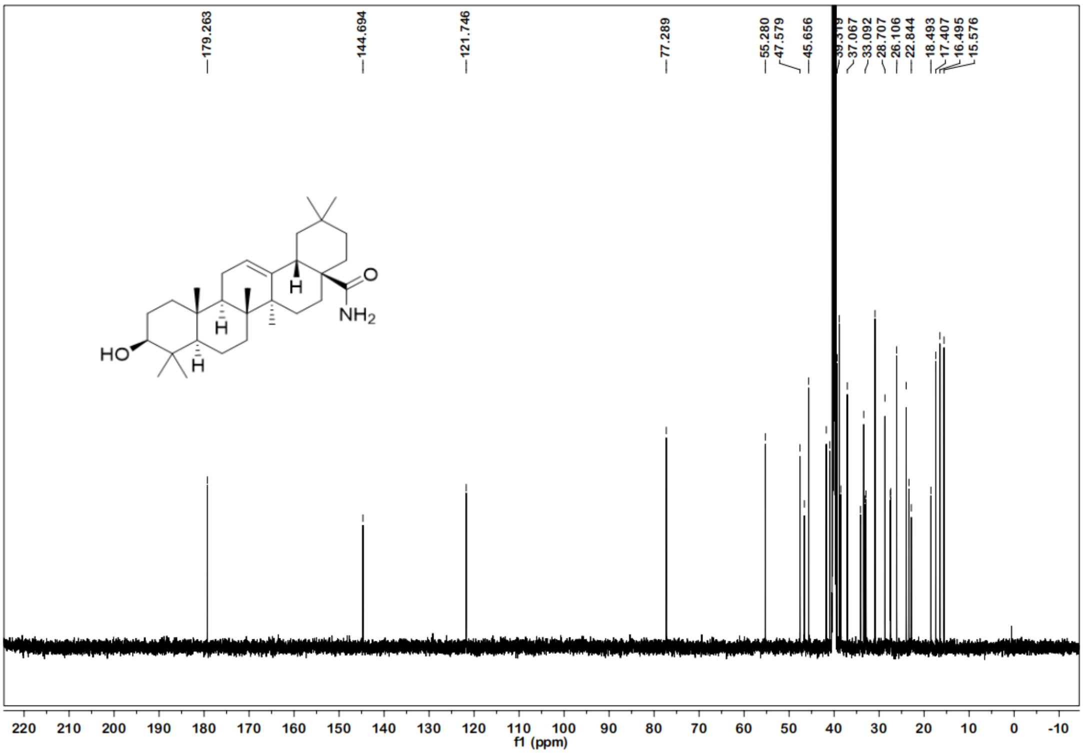
179.263
144.694
121.746
77.289
55.280
47.579
45.656
37.067
33.092
28.707
26.106
22.844
18.493
17.407
16.495
15.576
HO
H
NH2
O
f1 (ppm)

Compound **39**

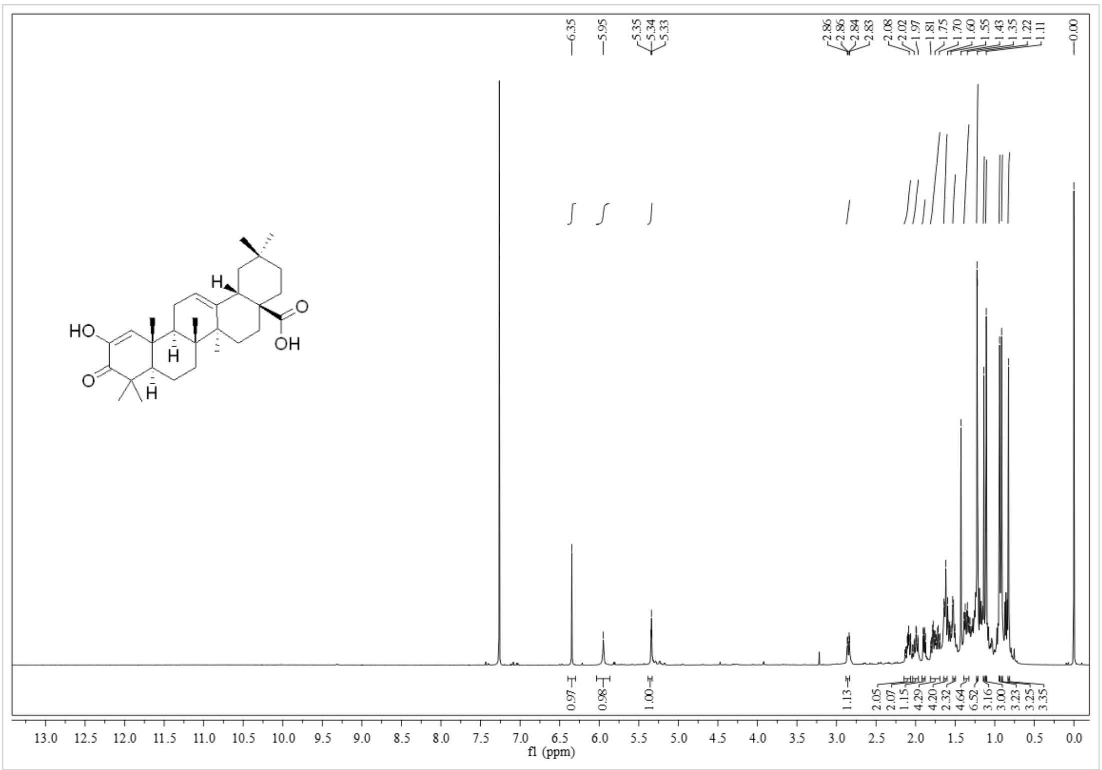
6.35
5.95
5.35
5.34
5.33
2.86
2.86
2.84
2.83
2.08
2.02
1.97
1.81
1.75
1.70
1.60
1.55
1.43
1.35
1.22
1.11
0.00
0.97
0.98
1.00
1.13
2.05
2.07
1.15
4.29
4.20
2.32
4.64
6.52
3.16
3.00
3.23
3.25
3.35
HO
O
H
OH
f1 (ppm)

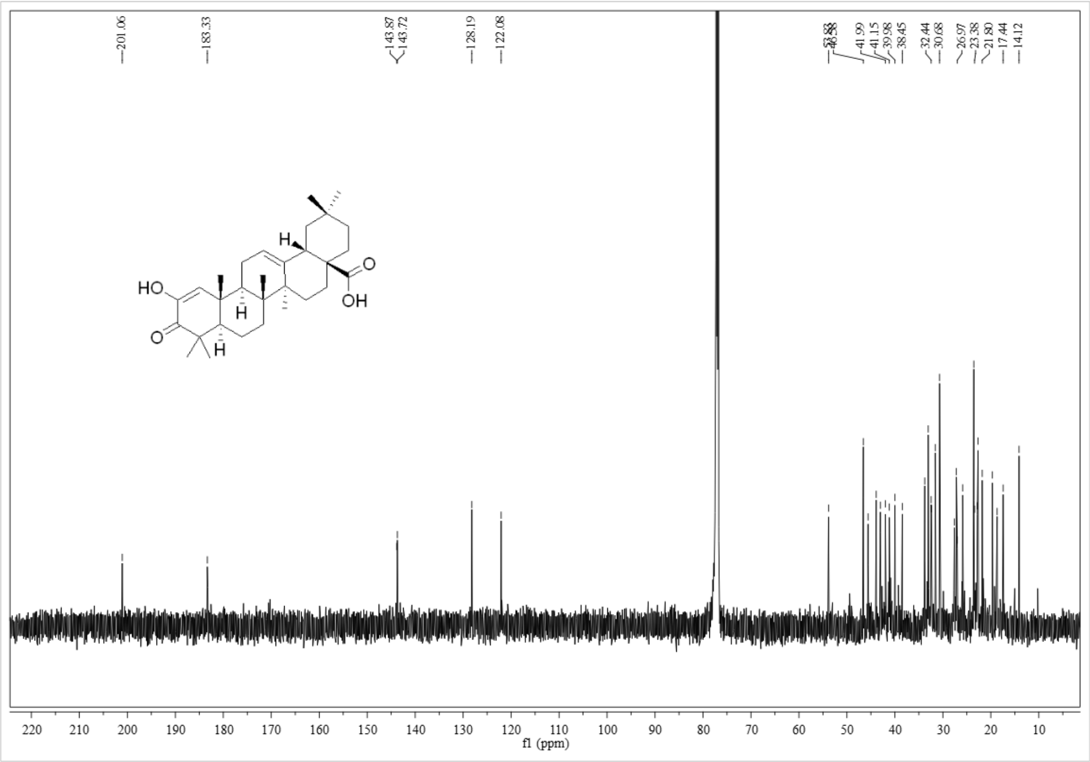

Compound **40**

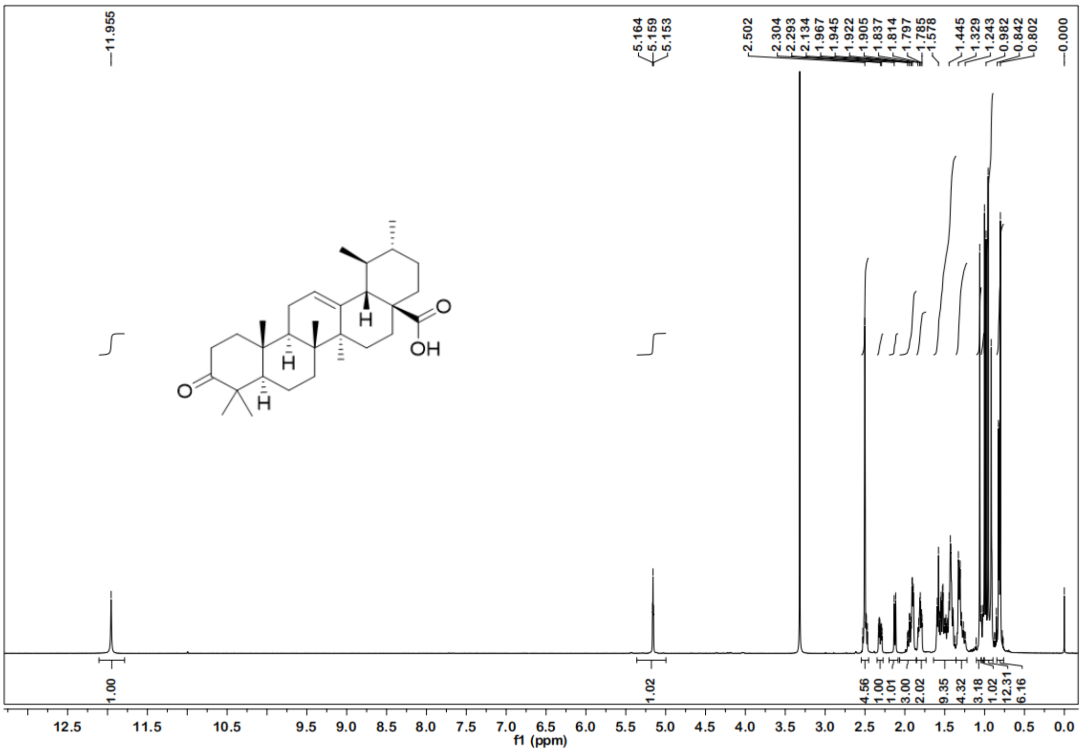

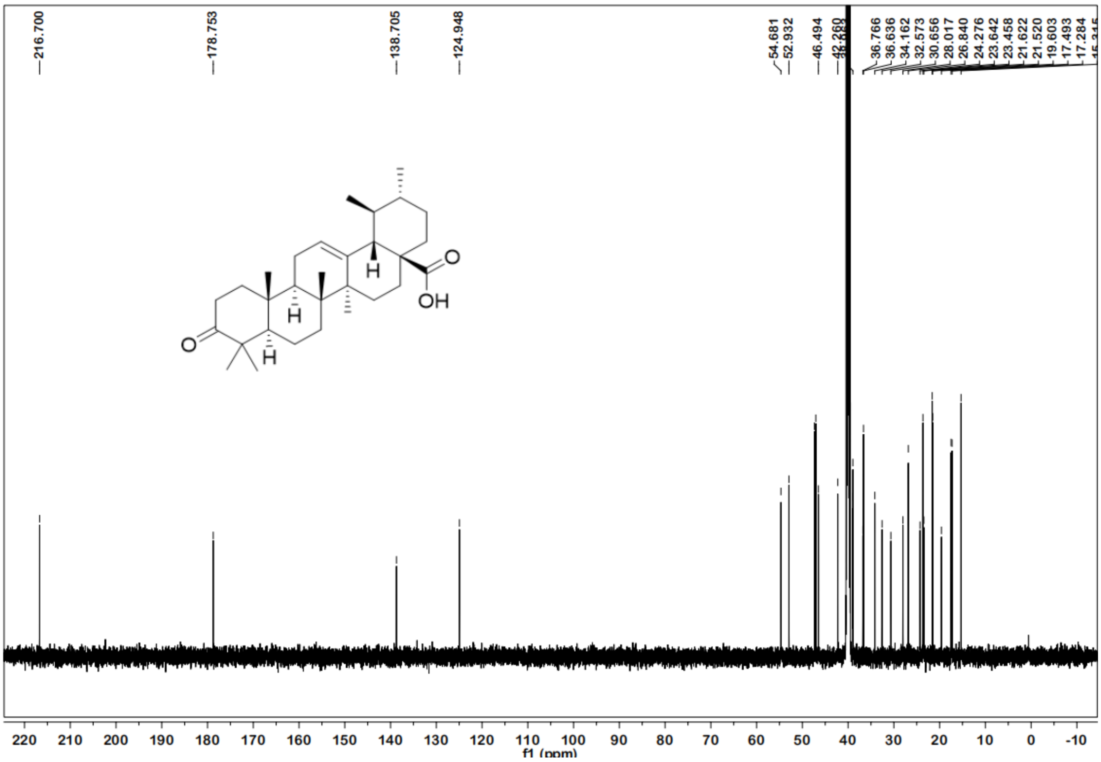

Compound **41**

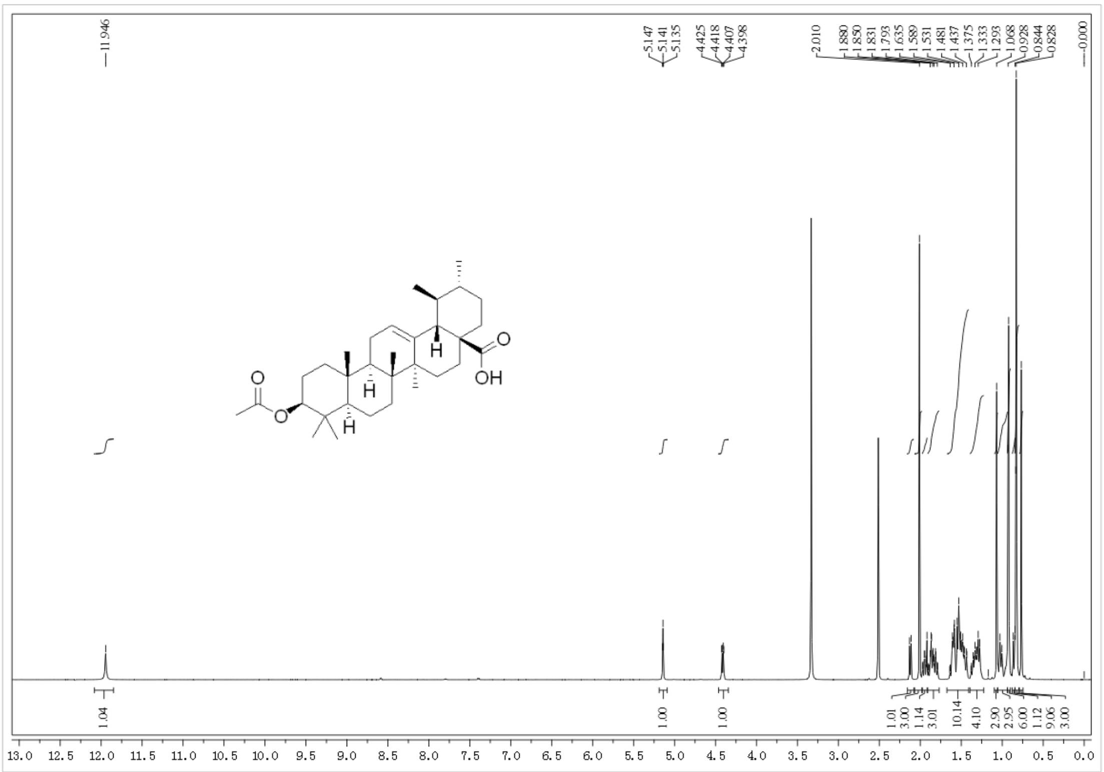

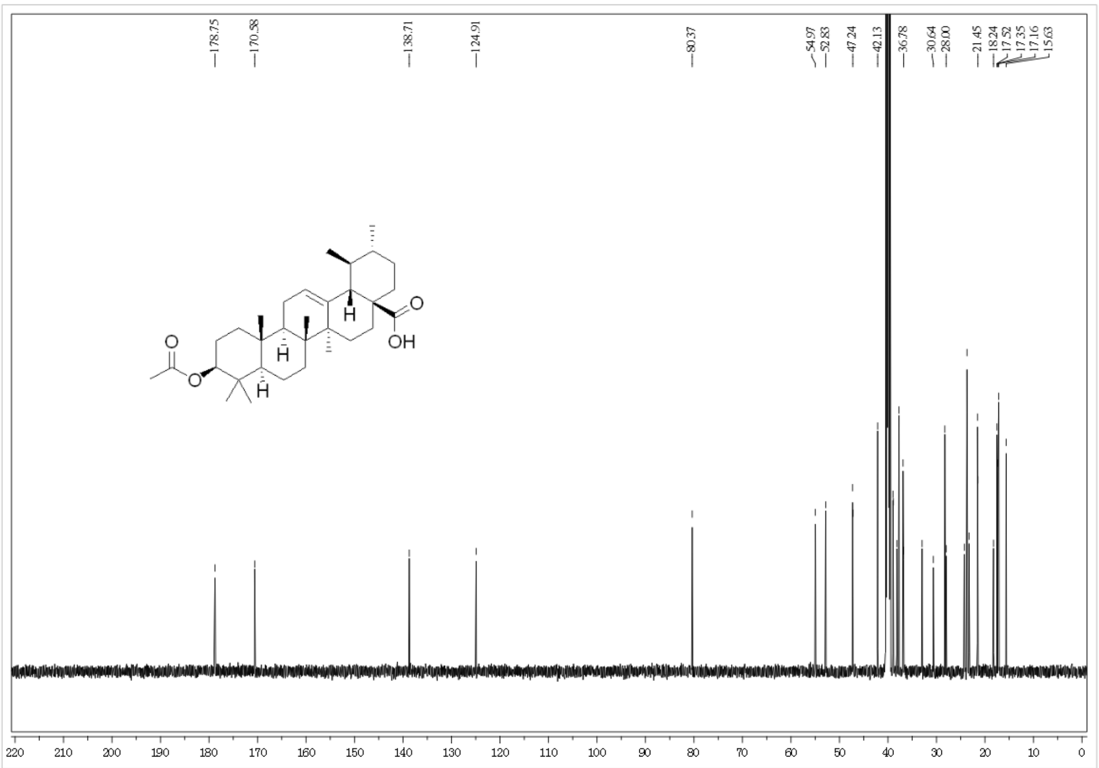

178.75
170.58
138.71
124.91
80.37
54.97
52.83
47.24
42.13
36.78
30.64
28.00
21.45
18.24
17.52
17.35
17.16
15.63
O
O
H
H
H
O
OH
220 210 200 190 180 170 160 150 140 130 120 110 100 90 80 70 60 50 40 30 20 10 0

Compound42

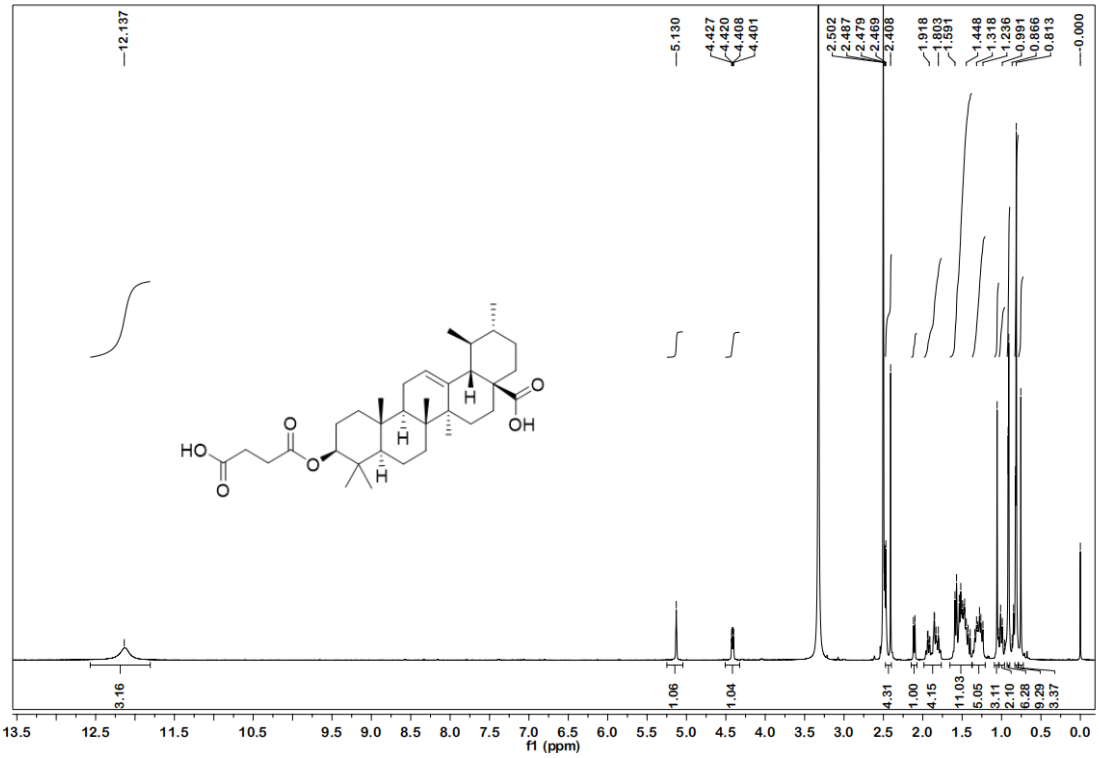

12.137
5.130
4.427
4.420
4.408
4.401
2.502
2.487
2.479
2.469
2.408
1.918
1.803
1.591
1.448
1.318
1.236
0.991
0.866
0.813
0.000
HO
O
O
O
H
H
H
O
OH
3.16
1.06
1.04
4.31
1.00
4.15
11.03
5.05
3.11
2.10
6.28
9.29
3.37
13.5 12.5 11.5 10.5 9.5 9.0 8.5 8.0 7.5 7.0 6.5 6.0 5.5 5.0 4.5 4.0 3.5 3.0 2.5 2.0 1.5 1.0 0.5 0.0
f1 (ppm)

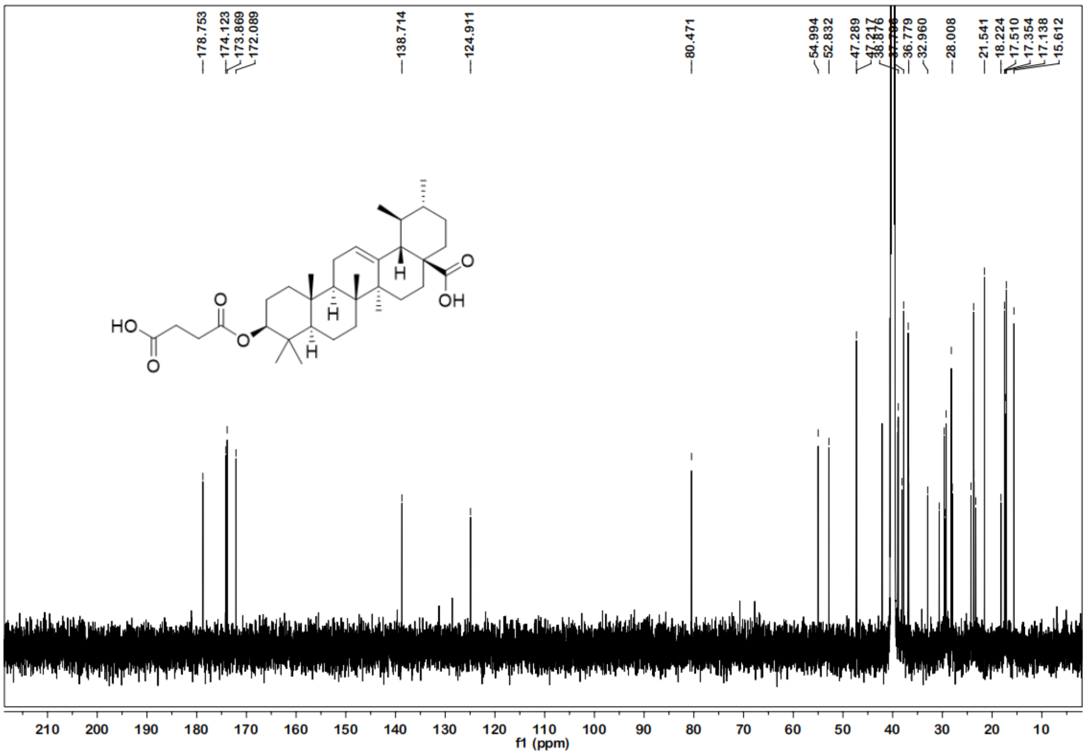

178.753
174.123
173.869
172.089
138.714
124.911
80.471
54.994
52.832
47.289
47.217
38.876
36.779
32.960
28.008
21.541
18.224
17.510
17.354
17.138
15.612
HO
O
OH
H
f1 (ppm)

Compound **43**

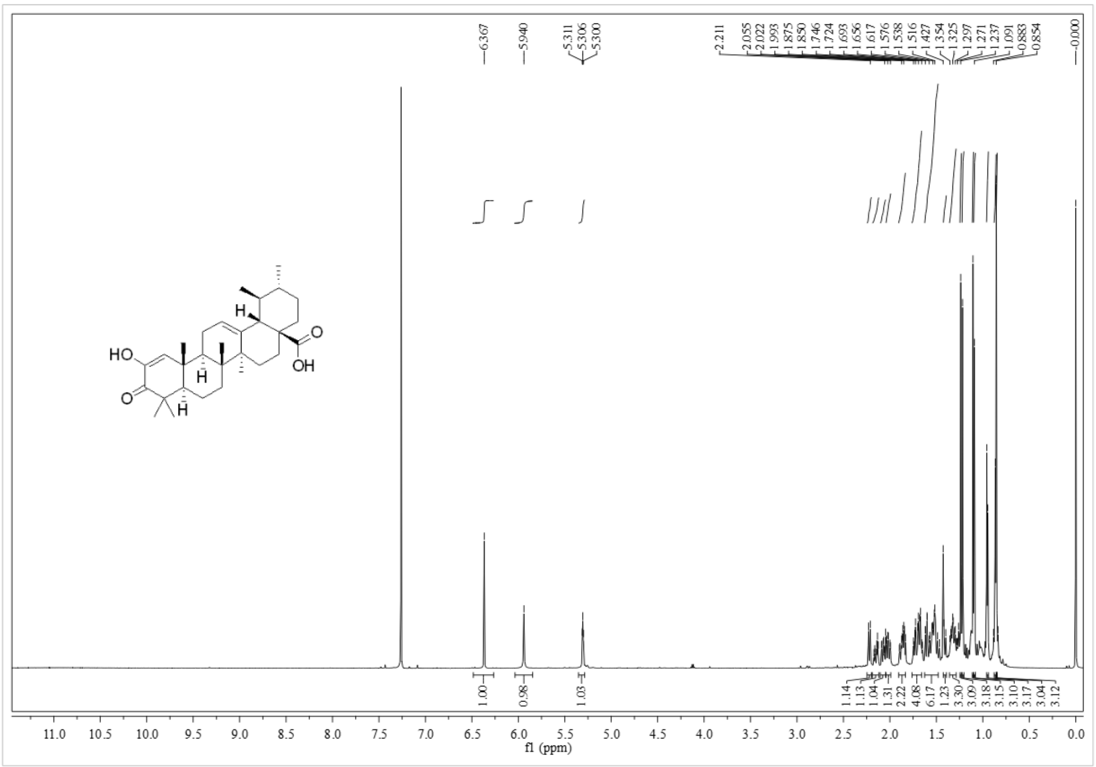

6.367
5.940
5.311
5.306
5.300
2.211
2.055
2.022
1.993
1.875
1.850
1.746
1.724
1.693
1.656
1.617
1.576
1.538
1.516
1.427
1.354
1.325
1.297
1.271
1.237
1.091
0.883
0.854
0.000
1.00
0.98
1.03
1.14
1.13
1.04
1.31
2.22
4.08
6.17
1.23
3.30
3.09
3.18
3.15
3.10
3.17
3.04
3.12
HO
O
OH
H
f1 (ppm)

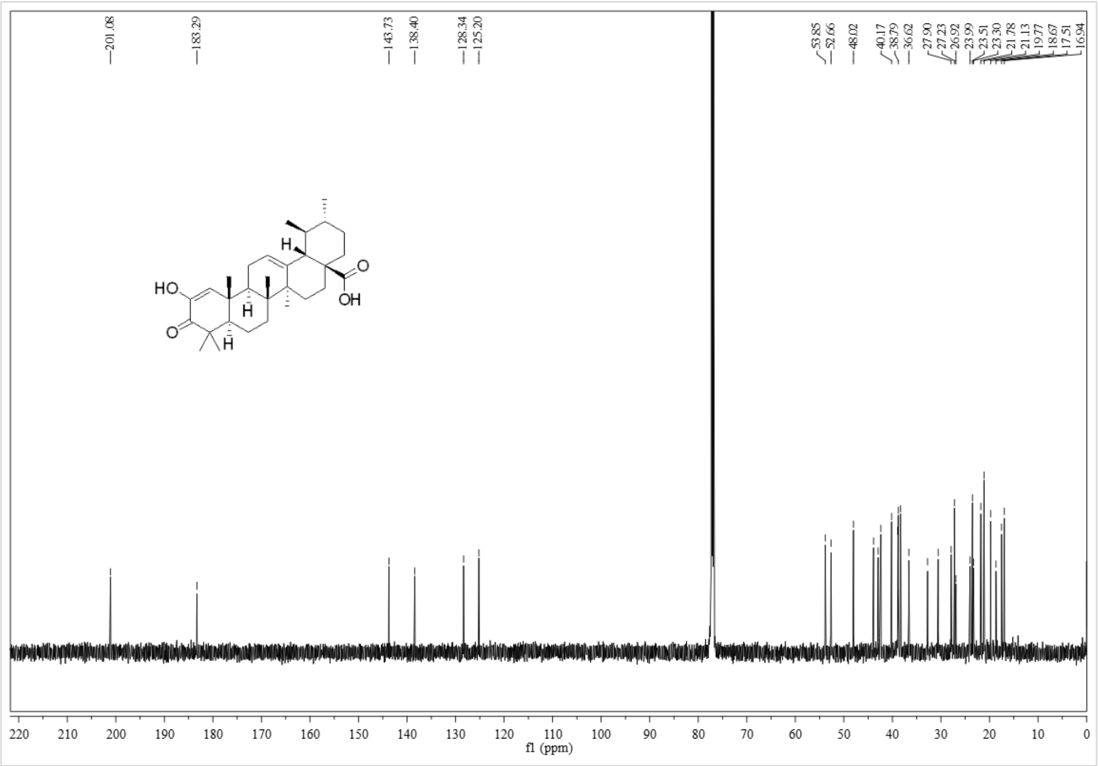

Reference

[1] L.W. Zou, T.Y. Dou, P. Wang, W. Lei, Z.M. Weng, J. Hou, D.D. Wang, Y.M. Fan, W.D. Zhang, G.B. Ge, L. Yang, Structure-Activity Relationships of Pentacyclic Triterpenoids as Potent and Selective Inhibitors against Human Carboxylesterase 1, Front Pharmacol, 8 (2017) 435.

[2] J.F. Li, Y. Zhao, M.M. Cai, X.F. Li, J.X. Li, Synthesis and evaluation of a novel series of heterocyclic oleanolic acid derivatives with anti-osteoclast formation activity, Eur J Med Chem, 44 (2009) 2796-2806.
